# SUMOylated Reptin maintains low intracellular ROS levels by activating mitophagy to drive gemcitabine resistance in gallbladder cancer

**DOI:** 10.1038/s41419-026-08598-0

**Published:** 2026-09-10

**Authors:** Chuanxin Yang, Wei Wang, Yaodi Shao, Xiangjun Wang, Yangming Liu, Liqin Yu, Sijie Zhang, Renhao Xue, Jian Wang

**Affiliations:** 1 Department of Hepatobiliary and Pancreatic Surgery, Shanghai Sixth People’s Hospital Affiliated to Shanghai Jiao Tong University School of Medicine, Shanghai, China; 2 Department of Biliary-Pancreatic Surgery, Renji Hospital Affiliated to Shanghai Jiao Tong University School of Medicine, Shanghai, China; 3 Shanghai Key Laboratory of Maternal Fetal Medicine, Shanghai Institute of Maternal-Fetal Medicine and Gynecologic Oncology, Clinical and Translational Research Center, Shanghai First Maternity and Infant Hospital, School of Medicine, Tongji University, Shanghai, China; 4 Department of Gynecology, Shanghai First Maternity and Infant Hospital, School of Medicine, Tongji University, Shanghai, China

**Keywords:** Oncogenes, Prognostic markers

## Abstract

Gemcitabine resistance remains a major challenge in the treatment of gallbladder cancer (GBC). Here, we elucidate a novel mechanism underlying gemcitabine resistance in GBC, centered on a self-reinforcing mitophagy/ROS/SENP3/Reptin loop. Gemcitabine-resistant GBC cells maintain significantly lower intracellular reactive oxygen species (ROS) levels than wild-type cells through enhanced mitophagy. This finding delineates a novel transcriptional mechanism that drives mitophagy under low ROS conditions. Mechanistically, this low ROS state decreases the protein abundance of the ROS sensor Sentrin/SUMO-specific protease 3 (SENP3), thereby promoting SUMOylation of its substrate, RuvB-like AAA+ ATPase 2 (Reptin), at the K456 site. SUMOylated Reptin translocates to the nucleus, where it acts as a transcriptional activator to specifically upregulate PTEN-induced putative kinase 1 (PINK1), a key mitophagy regulator. Enhanced PINK1 expression further amplifies mitophagy, effectively scavenging ROS and perpetuating the low ROS state that drives resistance, thus sustaining the gemcitabine-resistant phenotype. Clinically, low SENP3 expression and high Reptin expression correlate with poor gemcitabine response and shorter overall survival in GBC patients. Targeting this pathway, the Reptin ATPase inhibitor CB-6644 effectively suppressed PINK1 transcription, inhibited mitophagy, increased ROS accumulation, and reversed gemcitabine resistance both in vitro and in vivo. These findings identify the mitophagy/ROS/SENP3/Reptin loop as a core resistance mechanism in GBC, highlight SENP3 and Reptin as predictive biomarkers, and establish CB-6644 as a promising therapeutic agent to overcome gemcitabine resistance by disrupting this adaptive pathway.

## Introduction

Gallbladder cancer (GBC) is the most prevalent malignant tumor of the biliary tract and ranks sixth in incidence among gastrointestinal malignancies [1]. At present, radical resection is the only potentially curative approach for GBC. However, owing to the challenges in early diagnosis and the aggressive nature of the disease, approximately 80% of patients are diagnosed at an advanced stage [2], resulting in a low rate of radical resection and a dismal 5-year survival rate of only 5% to 18% [3, 4]. GBC typically exhibits “cold tumor” characteristics, with 80.4% of cases demonstrating a low tumor mutational burden. Furthermore, the incidence of actionable mutations in targets is low, conferring limited responsiveness to targeted therapies and immunotherapies [5]. As a result, chemotherapy remains the primary treatment modality for unresectable GBC, with gemcitabine recommended as a first-line agent according to NCCN guidelines [6, 7]. Nonetheless, a meta-analysis of 58 studies revealed that gemcitabine-based regimens achieve an objective response rate of only 23.9% in unresectable GBC, with median progression-free survival and overall survival (OS) of 5.1 months and 8.8 months, respectively [8]. Therefore, elucidating the mechanisms underlying gemcitabine resistance in GBC is urgently required to improve patient outcomes [9].

Gemcitabine, a cytidine analog, inhibits DNA synthesis by suppressing ribonucleotide reductase and competitively incorporating into DNA, thereby inducing DNA strand breaks. This process triggers substantial production of reactive oxygen species (ROS), ultimately resulting in oxidative stress-induced cell death [10]. Consequently, elevated intracellular ROS levels in tumor cells are critical for chemosensitivity [11]. However, this study demonstrates that, following gemcitabine treatment, intracellular ROS levels in gemcitabine-resistant GBC cells are significantly lower than those in wild-type (WT) cells. These findings suggest that reduced intracellular ROS levels after treatment may be a key driver of gemcitabine resistance in GBC. Therefore, it is essential to determine the underlying causes of low ROS levels in gemcitabine-resistant GBC cells following treatment and to investigate whether this phenomenon is associated with enhanced ROS-scavenging capacity. Elucidating this mechanism is vital for improving GBC sensitivity to gemcitabine.

As a key ROS-scavenging mechanism, mitophagy has attracted significant attention in tumor drug resistance research [12, 13]. Mitophagy is a selective autophagic process wherein damaged mitochondria, a primary source of intracellular ROS, are cleared under conditions of high intracellular ROS. This process reduces ROS levels, maintains intracellular redox homeostasis, and prevents cell death [14, 15]. Distinct from classical non-selective macroautophagy, the mechanisms and functions of mitophagy remain incompletely understood [16]. This study found that after gemcitabine treatment, gemcitabine-resistant GBC cells exhibit significantly stronger mitophagic activity than WT cells, potentially contributing to the observed low ROS levels and subsequent resistance. This phenomenon raises key questions: While high ROS levels typically trigger mitophagy, why is mitophagic activity stronger in gemcitabine-resistant GBC cells, which exhibit low ROS levels post-treatment, than in WT cells? Previous studies have confirmed that mitophagy can be activated through mechanisms independent of ROS. These include, for example, transcriptional regulation by factors such as Transcription Factor EB (TFEB) and Hypoxia-Inducible Factor 1 Alpha (HIF1A), as well as metabolic pathways involving AMP-activated protein kinase (AMPK), Ca^2+^, NAD^+^, and hypoxia-related signaling [16–18]. Therefore, whether similar mechanisms exist in gemcitabine-resistant cells with low ROS levels presents an innovative research question.

Whether low ROS-mediated mitophagy activation in gemcitabine-resistant GBC cells involves ROS sensors remains unclear. Sentrin/SUMO-specific protease 3 (SENP3), a member of the SENP family with deSUMOylation activity [18], functions as a ROS sensor: elevated ROS levels enhance its protein stability, leading to the accumulation of SENP3 [19–21]. Conversely, low ROS levels reduce SENP3 protein abundance. Thus, whether the sustained low ROS status in gemcitabine-resistant GBC cells activates mitophagy by downregulating SENP3 represents another key focus of this study.

SENP3 functions as both a ROS sensor and a deSUMOylase, exerting its functions by modulating the SUMOylation status of target proteins. Using mass spectrometry, this study identified RuvB-like AAA ATPase 2 (RUVBL2/Reptin) as a novel SENP3-binding protein. Reptin is an AAA+ ATPase involved in multiple biological processes, including transcriptional regulation, beyond its ATP hydrolysis activity [22]. The role and mechanisms of Reptin in GBC remain largely unexplored. SUMOylation is a reversible post-translational modification that regulates the subcellular localization, activity, and stability of target proteins [23]. Thus, whether Reptin undergoes SUMOylation, and if such modification affects its subcellular localization, activity, or stability—thereby activating mitophagy and contributing to gemcitabine resistance in GBC—warrants in-depth investigation.

This study found that gemcitabine-resistant GBC cells effectively limit excessive ROS accumulation by markedly enhancing mitophagy, maintaining intracellular ROS at low levels. This leads to decreased protein levels of the ROS sensor SENP3, which in turn increases SUMOylation of its target protein Reptin. SUMOylated Reptin was confirmed to transcriptionally upregulate PTEN-induced putative kinase 1 (PINK1), a core regulator of mitophagy, further enhancing mitophagic activity. Ultimately, this forms a self-reinforcing loop (mitophagy/ROS/SENP3/Reptin loop) that sustains the low ROS state and gemcitabine-resistant phenotype. This study not only uncovers a novel mechanism of tumor drug resistance wherein low ROS activates mitophagy, but also identifies potential new therapeutic targets.

## Results

### Low intracellular ROS levels post-treatment are crucial for gemcitabine resistance in GBC cells

**Fig. 1:**
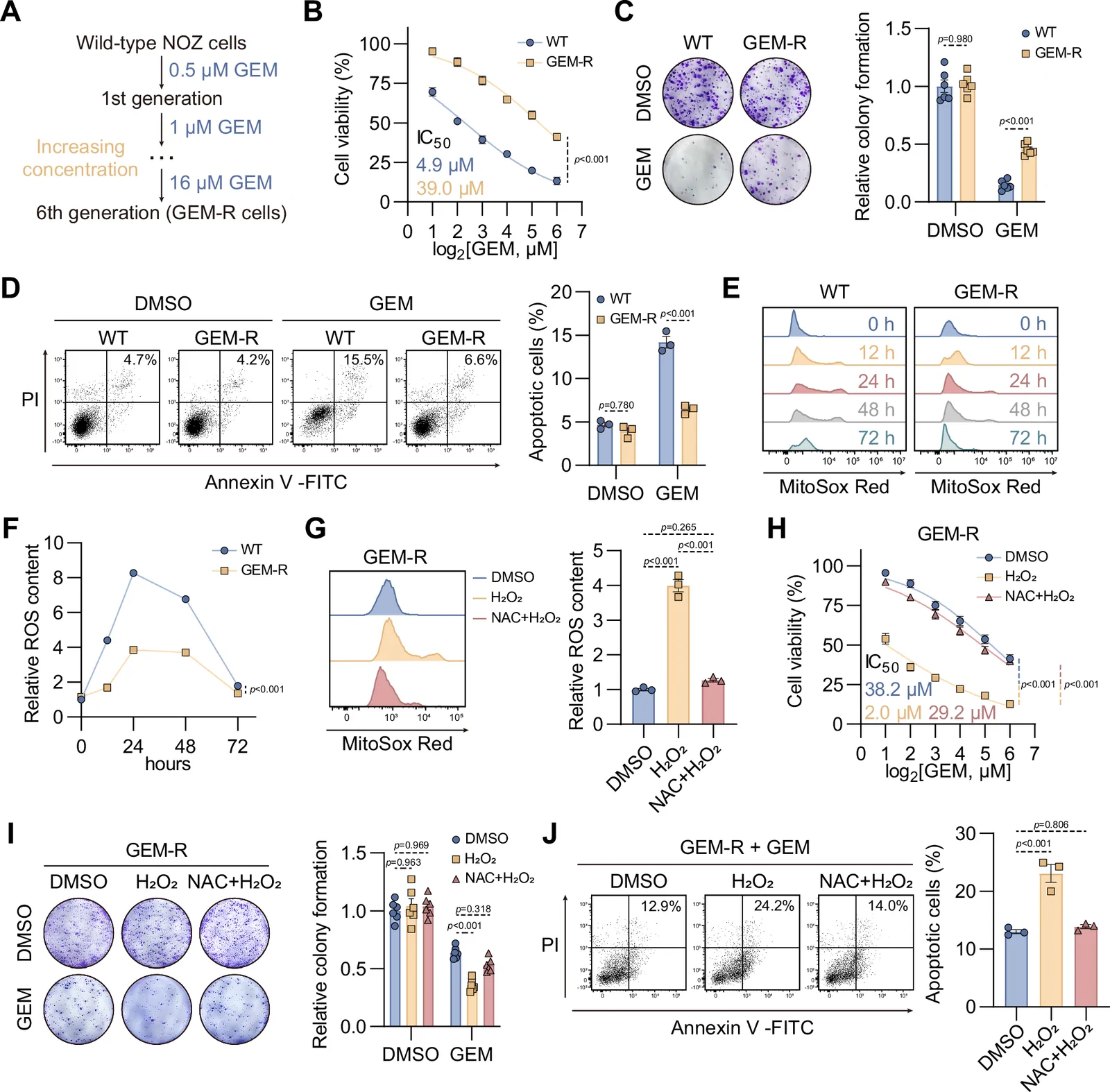
Low intracellular ROS levels post-treatment are crucial for gemcitabine resistance in GBC cells. **A** Schematic of the establishment of gemcitabine-resistant GBC cell line (GEM-R) using the concentration gradient escalation method. **B** Cell viability assays after 72 h of gemcitabine treatment. The half-maximal inhibitory concentration (IC50) of gemcitabine in GEM-R cells is significantly higher than in wild-type (WT) NOZ cells. **C** Colony formation assays after 96 h of treatment with 32 μM gemcitabine. GEM-R cells form significantly more colonies than WT NOZ cells. **D** Annexin V-FITC/PI staining followed by flow cytometry analysis after 72 h of treatment with 4 μM gemcitabine. The apoptosis rate of GEM-R cells is significantly lower than that of WT NOZ cells. **E**, **F** Detection of intracellular ROS levels (MitoSOX Red staining, flow cytometry) revealed that while basal ROS levels were higher in GEM-R cells, gemcitabine treatment induced a temporal response (peaking at 24 h) after which ROS levels remained significantly lower in GEM-R cells than in WT cells throughout the treatment period. **G** MitoSOX Red staining to detect the effect of exogenous H_2_O_2_ (100 μM) and antioxidant N-acetylcysteine (NAC, 5 mM) on intracellular ROS levels in GEM-R cells. **H** Cell viability assays to evaluate the effect of H_2_O_2_ and NAC on gemcitabine sensitivity in GEM-R cells. H_2_O_2_ treatment significantly reduces the gemcitabine IC50 in GEM-R cells, while NAC antagonizes this effect. **I** Colony formation assays after 96 h of treatment with 32 μM gemcitabine (in combination with H_2_O_2_/NAC). H_2_O_2_ treatment significantly reduces colony formation in GEM-R cells, an effect reversed by NAC. **J** Apoptosis detection after 72 h of treatment with 32 μM gemcitabine (in combination with H_2_O_2_/NAC). H_2_O_2_ treatment significantly increases the apoptosis rate of GEM-R cells, an effect antagonized by NAC. Data represent the mean ± SEM (*n* = 3 independent experiments in (**B**, **D**, **F**, **G**, **H**, **J**); *n* = 6 independent experiments in **C**, **I**). Two-way ANOVA (**B**, **F**, **H**) and one-way ANOVA (**C**, **D**, **G**, **I**, **J**) were used.

To investigate gemcitabine resistance mechanisms, we successfully established a gemcitabine-resistant GBC cell line (GEM-R) using a concentration gradient escalation method. Starting with WT NOZ cells as the parental line, cells were initially exposed to 500 nM gemcitabine. After surviving cells recovered to the logarithmic growth phase, they were passaged, and the drug concentration was incrementally increased to 1 μM. This cycle was repeated, with cells ultimately reaching and maintaining growth in 16 μM gemcitabine by week 24 (Fig. 1A). To verify the resistance of GEM-R cells, CCK-8 cell viability assays showed that the half-maximal inhibitory concentration (IC50) of gemcitabine in GEM-R cells was significantly higher than in WT NOZ cells (Fig. 1B). Colony formation assays confirmed that, under treatment with the same gemcitabine concentration, GEM-R cells formed significantly more colonies than WT cells (Fig. 1C). Annexin V-FITC/PI staining combined with flow cytometric analysis revealed that the apoptosis rate of GEM-R cells was significantly lower than that of WT NOZ cells after gemcitabine treatment (Fig. 1D).

To explore whether resistance correlates with ROS levels, we measured intracellular ROS levels under basal conditions and after gemcitabine treatment using the fluorescent probe MitoSOX Red [24]. Results showed that following gemcitabine treatment (which induced a transient ROS peak at 24 h before declining), ROS levels were significantly lower in GEM-R cells than in WT cells throughout the observation period, whereas basal levels showed no significant difference (Fig. 1E, F), indicating that gemcitabine-resistant GBC cells possess enhanced ROS-scavenging capacity. Further rescue experiments demonstrated that exogenous hydrogen peroxide (H_2_O_2_)-induced elevation of intracellular ROS in GEM-R cells significantly enhanced their sensitivity to gemcitabine. Conversely, treatment with the antioxidant N-acetylcysteine (NAC), which abrogated the ROS-elevating effect of H_2_O_2_, restored the drug resistance of GEM-R cells (Fig. 1G–J). Taken together, these results indicate that maintaining low intracellular ROS levels after gemcitabine treatment is a critical factor in GBC cells acquiring resistance, and exogenous elevation of ROS levels can effectively reverse this resistance, enhancing gemcitabine sensitivity.

### Role of ROS sensor SENP3 in gemcitabine resistance of GBC cells

**Fig. 2:**
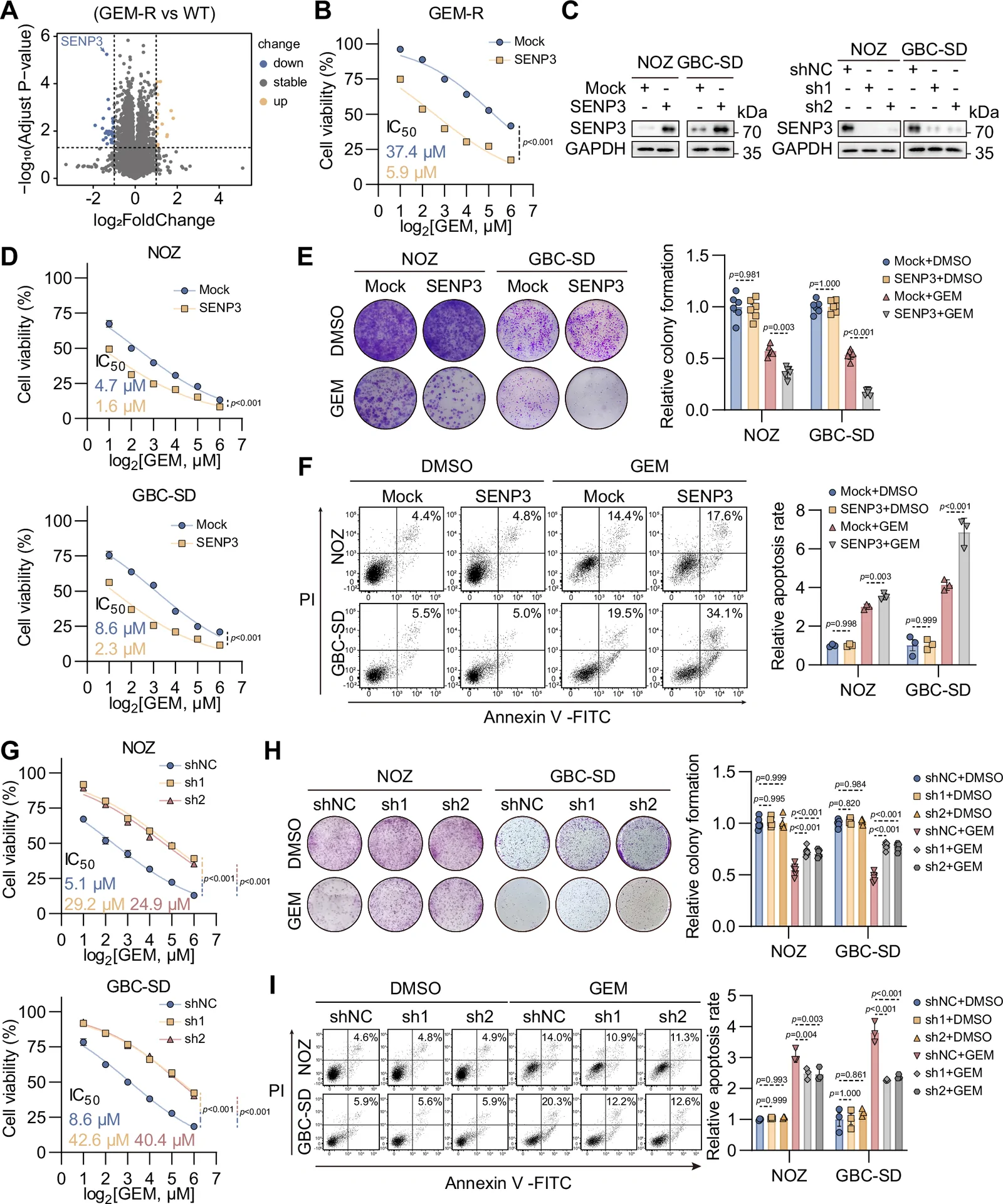
The ROS sensor SENP3 enhances gemcitabine sensitivity in GBC cells in vitro. **A** Volcano plot of differentially expressed proteins between gemcitabine-resistant GBC cell line (GEM-R) and wild-type (WT) NOZ cells after gemcitabine treatment (based on proteomic analysis). Screening criteria: *p* < 0.05, fold change < 0.5 or > 2. **B** Cell viability assays after 72 h of gemcitabine treatment. SENP3-overexpression in GEM-R cells significantly reduces their half-maximal inhibitory concentration (IC50) of gemcitabine. **C** Western blot validation of the efficiency of stable SENP3-overexpression or SENP3-knockdown in GBC cells (NOZ, GBC-SD). **D** Cell viability assays after 72 h of gemcitabine treatment. SENP3-overexpression significantly reduces the gemcitabine IC50 in GBC cells (NOZ, GBC-SD). **E** Colony formation assays after 96 h of gemcitabine treatment. SENP3-overexpression significantly reduces the number of colonies formed by NOZ and GBC-SD cells. **F** Annexin V-FITC/PI staining followed by flow cytometry analysis after 72 h of gemcitabine treatment. SENP3-overexpression significantly increases the apoptosis rate of NOZ and GBC-SD cells. **G** Cell viability assays after 72 h of gemcitabine treatment. SENP3-knockdown significantly increases the gemcitabine IC50 in NOZ and GBC-SD cells. **H** Colony formation assays after 96 h of gemcitabine treatment. SENP3-knockdown significantly increases the number of colonies formed by NOZ and GBC-SD cells. **I** Annexin V-FITC/PI staining followed by flow cytometry analysis after 72 h of gemcitabine treatment. SENP3-knockdown significantly reduces the apoptosis rate of NOZ and GBC-SD cells. Data represent the mean ± SEM (*n* = 3 independent experiments in (**A**, **B**, **D**, **F**, **G**, **I**); *n* = 6 independent experiments in **E**, **H**). Two-way ANOVA (**B**, **D**, **G**) and one-way ANOVA (**E**, **F**, **H**, **I**) were used.

To elucidate the specific mechanism by which low ROS levels mediate gemcitabine resistance, we performed global proteomic analysis on WT NOZ and GEM-R cells following gemcitabine treatment (*n* = 3 biological replicates per group). Using stringent screening criteria (*p* < 0.05, fold change < 0.5 or > 2), we found that SENP3 protein expression was most significantly downregulated in GEM-R cells after gemcitabine stimulation (Fig. 2A), a finding confirmed by Western blot (Supplementary Fig. 1A). To verify whether SENP3 senses ROS levels in GBC cells, Western blot showed that exogenous H_2_O_2_ treatment increased SENP3 protein levels in GEM-R cells in a concentration-dependent manner, an effect antagonized by the antioxidant NAC (Supplementary Fig. 1B). The same trend was observed in WT NOZ cells (Supplementary Fig. 1C). Consistent with previous reports [20], we confirmed that ROS enhances SENP3 protein stability not via transcriptional regulation but by inhibiting its proteasomal degradation (Supplementary Fig. 1D, E). These results confirm that SENP3 functions as a ROS sensor in GBC cells.

To further explore the regulatory role of SENP3 in drug resistance, transient overexpression of SENP3 in GEM-R cells significantly enhanced their sensitivity to gemcitabine (Fig. 2B). Subsequently, we established stable SENP3-overexpression and SENP3-knockdown cell lines in NOZ and GBC-SD cells (efficiency verified by Western blot; Fig. 2C, Supplementary Fig. 1F). Functional assays consistently demonstrated that SENP3-overexpression significantly enhanced gemcitabine sensitivity in GBC cells (reduced cell viability, decreased colony formation, and increased apoptosis; Fig. 2D–F), while SENP3-knockdown significantly enhanced gemcitabine resistance (increased cell viability, enhanced colony formation, and reduced apoptosis; Fig. 2G–I). Notably, SENP3-overexpression had no significant effect on the basal proliferation or migration of GBC cells (Supplementary Fig. 1G–1H).

**Fig. 3:**
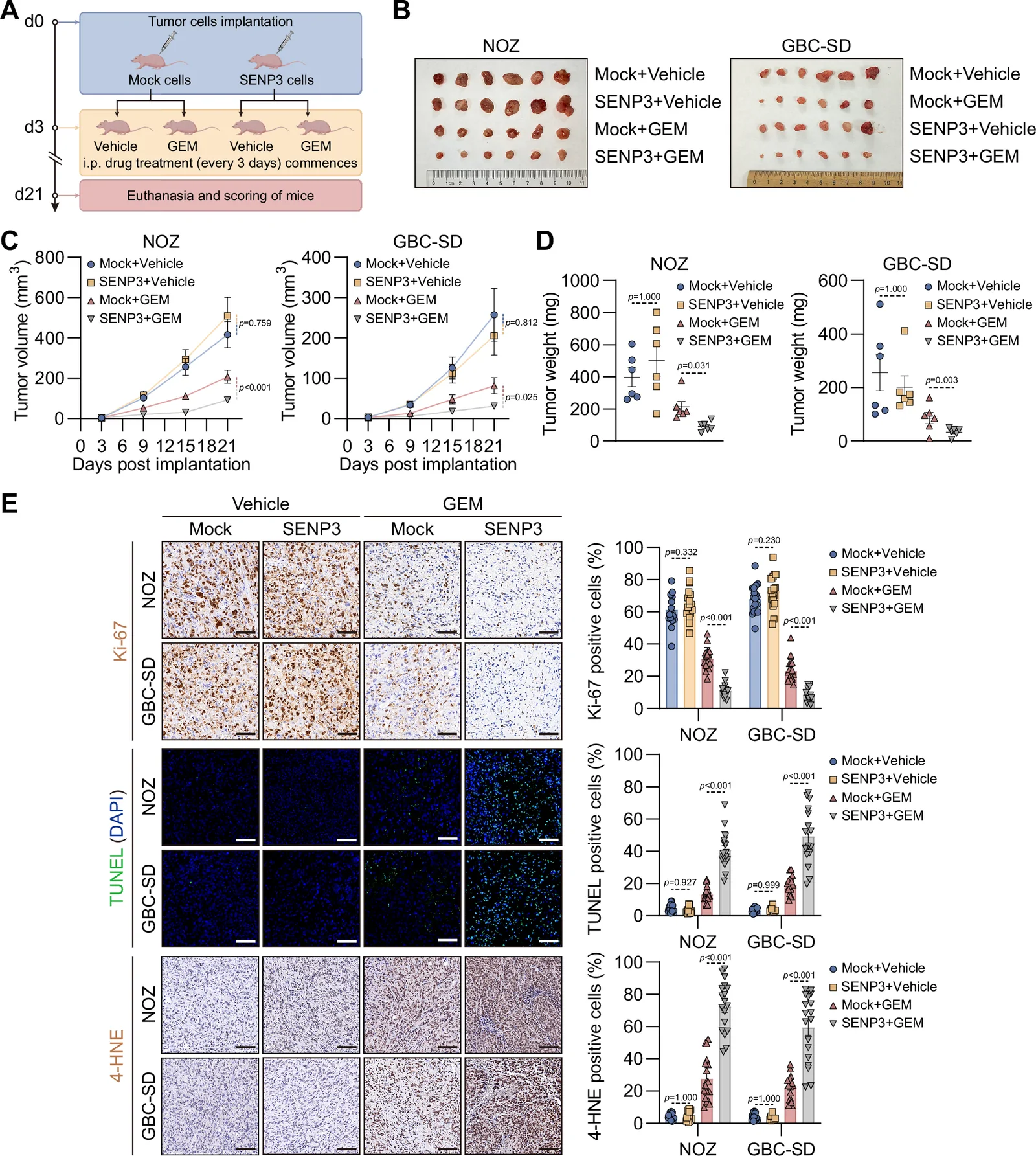
SENP3 enhances gemcitabine sensitivity in GBC cells in vivo. **A** Schematic of the xenograft tumor experiment in BALB/c nude mice. 2 × 10^6^ NOZ or GBC-SD cells were injected subcutaneously into the right axilla of athymic nude mice. **B** Gross photographs of xenograft tumors in each group at the endpoint of treatment. **C**, **D** In the gemcitabine treatment groups, tumor volume (**C**) and weight (**D**) in the SENP3-overexpression group are significantly smaller than in the empty vector group. In the saline control groups, there are no significant differences in tumor volume or weight between the SENP3-overexpression and empty vector groups. **E** Staining analysis of tumor tissue sections (scale bar: 100 μm). Ki-67 staining for proliferation, TUNEL staining for apoptosis, and 4-HNE staining for oxidative damage. In the gemcitabine treatment groups, compared with the empty vector group, tumors in the SENP3-overexpression group exhibit a significantly reduced proliferation index, significantly increased apoptosis rate, and significantly enhanced oxidative damage. In the saline control groups, there are no significant differences in these indices between the two groups. Data represent the mean ± SEM (*n* = 6 animals per group). Two-way ANOVA (**C**), Kruskal-Wallis test (**D**), and one-way ANOVA (**E**) were used.

To systematically evaluate the role of SENP3 in gemcitabine sensitivity of GBC, we established a xenograft model using BALB/c nude mice (Fig. 3A). GBC cells (NOZ and GBC-SD) stably overexpressing either empty vector (Mock) or SENP3 were subcutaneously inoculated into the right flank of nude mice. After tumor formation, animals were randomly assigned to receive either gemcitabine or saline (vehicle) via intraperitoneal injection. Dynamic monitoring showed that in the saline treatment groups, tumor growth curves of the SENP3-overexpression and Mock groups overlapped closely, with no significant differences in endpoint tumor volume or weight, indicating that SENP3 overexpression itself has no autonomous effect on in vivo tumor proliferation (Fig. 3B–D). However, in the gemcitabine treatment groups, tumor volume and weight in the SENP3-overexpression group were significantly smaller than those in the Mock group (Fig. 3B–D). Further analysis using Ki-67 staining (proliferation), TUNEL staining (apoptosis), and staining for the lipid peroxidation product 4-HNE (oxidative damage) showed that in the saline treatment groups, there were no significant differences in proliferation index, apoptosis rate, or oxidative damage level between groups. In the gemcitabine treatment groups, compared with the Mock group, the SENP3-overexpression group exhibited a significantly reduced proliferation index, a significantly increased apoptosis rate, and significantly enhanced oxidative damage (Fig. 3E).

Taken together, these in vitro and in vivo findings confirm that SENP3, as a ROS sensor, significantly enhances gemcitabine sensitivity in GBC.

### SENP3 inhibits Reptin nuclear translocation via deSUMOylation

**Fig. 4:**
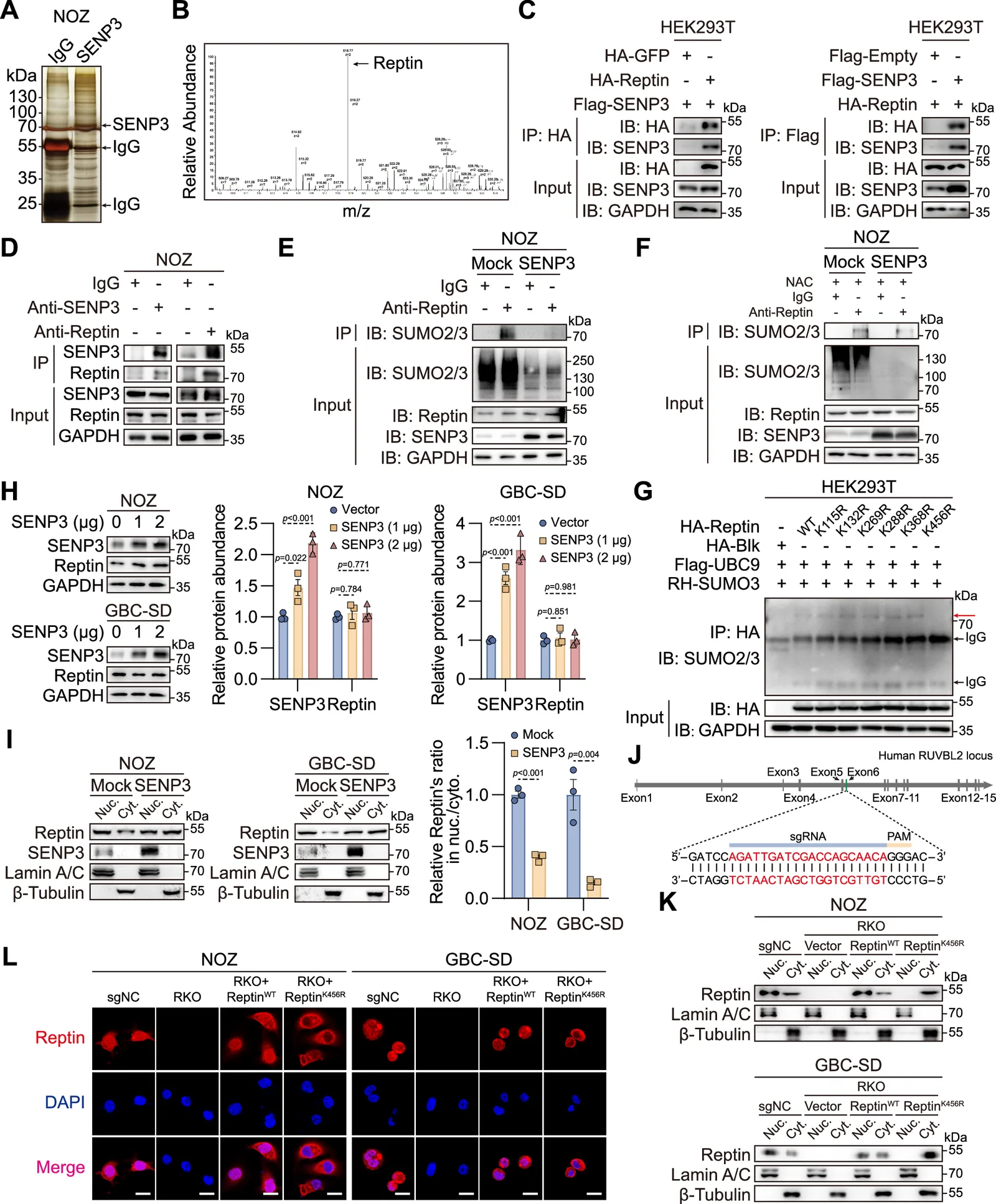
SENP3 inhibits Reptin nuclear localization via deSUMOylation. **A** SDS-PAGE silver staining analysis of SENP3 co-immunoprecipitation (co-IP) products in NOZ cells. Specific bands are only present in the SENP3 antibody group, not in the IgG control group. **B** Mass spectrometry analysis of SENP3 co-IP products. Reptin peptides are significantly enriched. **C**, **D** Reciprocal validation of the interaction between SENP3 and Reptin. **C** Exogenous co-IP (293 T cells transfected with indicated plasmids for 72 h); (**D**) Endogenous co-IP (NOZ cells). Western blot confirms specific binding between SENP3 and Reptin. **E** SUMOylation of Reptin and regulation by SENP3. Denaturing Reptin co-IP in NOZ cells with SENP3-overexpression or empty vector. Detection with SUMO2/3 antibody shows: a SUMOylated Reptin band is present in the empty vector group, while this modification is absent in the SENP3 overexpression group. **F** Denaturing Reptin co-IP performed in NOZ cells under conditions of maintained ROS levels (via NAC). Detection with SUMO2/3 antibody confirms that SENP3 overexpression removes the SUMOylated Reptin band, even under constant ROS. **G** Identification of the critical SUMOylation site in Reptin. Denaturing co-IP after transfection of 293 T cells with wild-type (WT) or mutant Reptin plasmids for 72 h. Detection with SUMO2/3 antibody shows: SUMOylation is completely absent only in the K456R mutant (red arrow). **H** Effect of SENP3-overexpression on Reptin protein stability. Western blot analysis of NOZ/GBC-SD cells transfected with gradient concentrations of SENP3 plasmid for 72 h. Total Reptin protein levels show no significant change. **I** Regulation of Reptin subcellular localization by SENP3 (nuclear-cytoplasmic fractionation). SENP3-overexpression reduces the nuclear fraction and increases the cytoplasmic fraction of Reptin (NOZ/GBC-SD cells). **J** Schematic of CRISPR-Cas9-mediated Reptin knockout (RKO) strategy (targeting exon 6). **K**, **L** Critical role of K456 SUMOylation in Reptin nuclear localization (rescue experiments in RKO cells). **K** Nuclear-cytoplasmic fractionation Western blot; (**L**) Immunofluorescence staining (scale bar: 25 μm). Rescued WT Reptin localizes to both nucleus and cytoplasm, while the K456R mutant is restricted to the cytoplasm. Data represent the mean ± SEM (*n* = 3 independent experiments). One-way ANOVA (**H**) and Student’s t-tests (**I**) were used.

Given that SENP3 functions as a deSUMOylase, its regulation of gemcitabine resistance likely relies on the deSUMOylation of a specific target protein. To test this hypothesis, we performed SENP3 co-immunoprecipitation (co-IP) in NOZ cells. SDS-PAGE silver staining revealed specific bands in the SENP3 antibody group, absent in the IgG control group (Fig. 4A). Mass spectrometry analysis identified the AAA+ ATPase Reptin as the most prominent SENP3-interacting protein (Fig. 4B, Supplementary Fig. 2A). Reciprocal exogenous co-IP (Fig. 4C) and reciprocal endogenous co-IP (Fig. 4D) experiments confirmed the physical interaction between SENP3 and Reptin.

To verify that SENP3 mediates Reptin deSUMOylation, we performed denaturing co-IP (to expose modified epitopes) using Reptin antibody in NOZ cells transfected with empty vector or SENP3-overexpressing constructs. Detection with SUMO2/3 antibody (SENP3 specifically targets SUMO2/3, not SUMO1) showed a distinct SUMOylated Reptin band in the empty vector group, which was absent in the SENP3-overexpression group (Fig. 4E), indicating endogenous Reptin SUMOylation that SENP3 specifically removes. We repeated the denaturing co-IP under conditions where ROS levels were maintained constant by NAC, which confirmed that SENP3 directly modulates Reptin SUMOylation independent of ROS signaling (Fig. 4F).

To identify the specific SUMOylation site, we used bioinformatics tools SUMOplot and GPS-SUMO to predict six potential SUMOylation sites: K115, K132, K269, K288, K368, and K456 (Supplementary Fig. 2B). We constructed plasmids encoding mutants with lysine (K) → arginine (R, cannot be SUMOylated) substitutions. Denaturing co-IP after transfection into 293 T cells showed that the SUMOylation band was completely abolished only in the Reptin K456R mutant (Fig. 4G), confirming K456 as the critical SUMOylation site. Notably, the K456R mutation did not affect the SENP3-Reptin interaction (Supplementary Fig. 2C).

As SUMOylation regulates subcellular localization, activity, or stability [23]. We first examined Reptin protein levels in SENP3-overexpressing GBC cells. SENP3-overexpression did not affect total Reptin protein levels (Fig. 4H), indicating that SUMOylation does not regulate its stability. Nuclear-cytoplasmic fractionation assays showed that SENP3-overexpression reduced nuclear Reptin distribution and increased cytoplasmic distribution (Fig. 4I). We used CRISPR-Cas9 technology to generate Reptin-knockout (RKO) NOZ and GBC-SD cells (Fig. 4J, Supplementary Fig. 2D). Subsequent rescue experiments in RKO cells, where WT Reptin or the K456R mutant was reintroduced, followed by nuclear-cytoplasmic fractionation assays, revealed that rescued WT Reptin localized to both the nucleus and cytoplasm, whereas the rescued K456R mutant was restricted to the cytoplasm (Fig. 4K). Immunofluorescence staining confirmed these subcellular localization differences (Fig. 4L).

Taken together, our findings demonstrate that SUMOylation of Reptin at the critical K456 site determines its nuclear localization, and SENP3 specifically inhibits Reptin nuclear translocation by catalyzing its deSUMOylation.

### SUMOylated Reptin enhances gemcitabine resistance in GBC by transcriptionally upregulating the key mitophagy gene PINK1

To clarify the relationship between Reptin and gemcitabine resistance, cell viability assays showed that RKO cells exhibited significantly higher gemcitabine sensitivity compared to control (sgNC) cells. Rescue experiments in RKO cells revealed that reconstitution with WT Reptin enhanced gemcitabine resistance, whereas reconstitution with the K456R mutant had no such effect (Supplementary Fig. 3A), indicating that SUMOylated Reptin is critical for gemcitabine resistance in GBC.

**Fig. 5:**
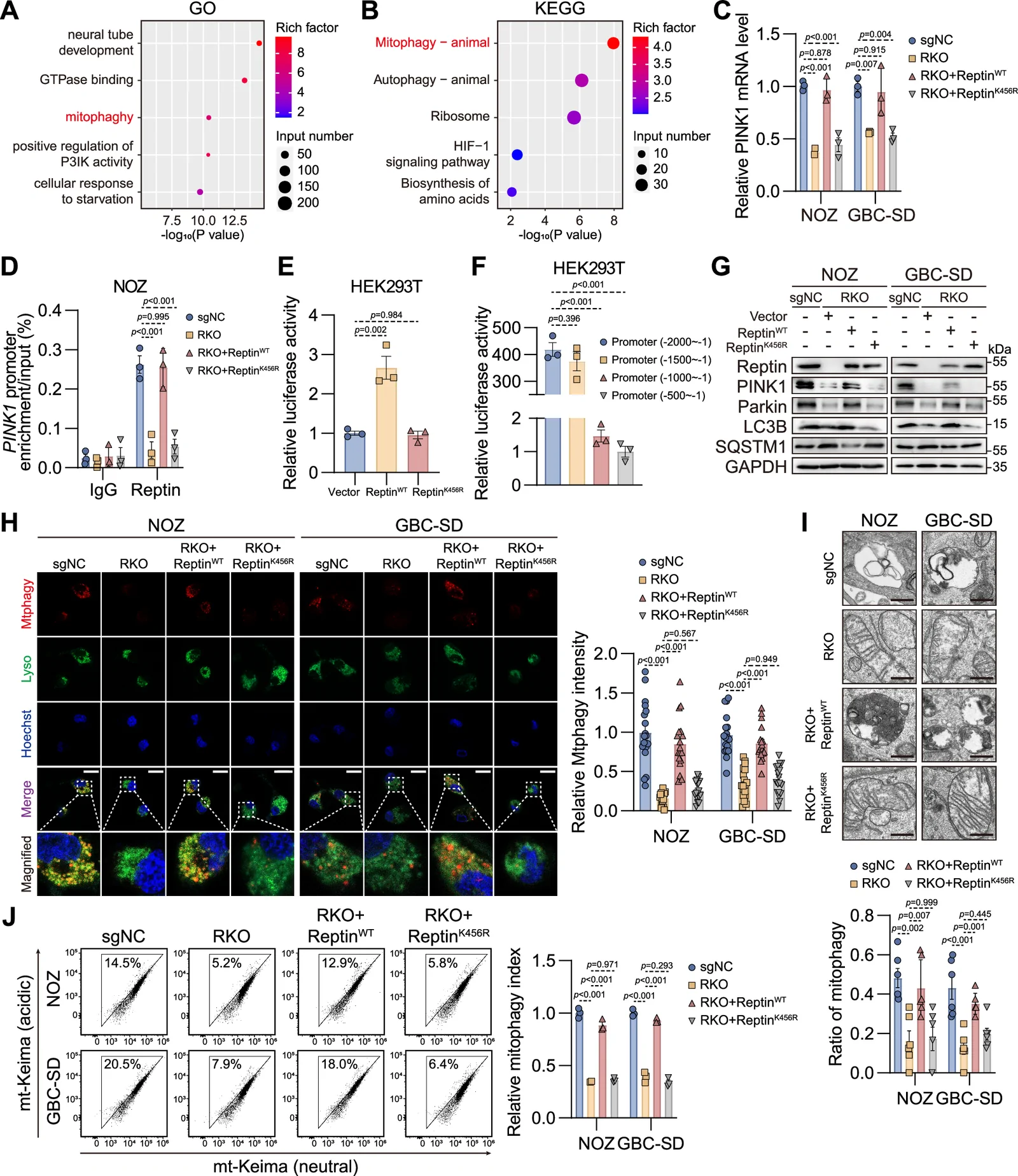
SUMOylated Reptin promotes mitophagy through transcriptional activation of PINK1. **A**, **B** Bubble plots of (**A**) GO and (**B**) KEGG enrichment analyses of genes associated with ChIP-seq peaks (the mitophagy pathway is significantly enriched). **C** RT-qPCR detection of PINK1 mRNA expression in NOZ and GBC-SD cells after 48 h of gemcitabine treatment. Reptin-knockout significantly reduces PINK1 levels; this effect is reversed by rescue with wild-type (WT) Reptin but not with the SUMOylation-deficient mutant (K456R). **D** ChIP-qPCR analysis in NOZ cells. Knockdown of Reptin reduces its enrichment at the PINK1 promoter. This enrichment is restored by reconstitution with WT Reptin, but not with the K456R SUMOylation-deficient mutant. **E** Dual-luciferase reporter assay. Overexpression of WT Reptin significantly activates transcription of the reporter gene containing the PINK1 promoter (−2 kb), while the K456R mutant has no such effect. **F** Dual-luciferase reporter assays were performed using serially truncated PINK1 promoter constructs (lengths: −2 kb, −1.5 kb, −1 kb, −0.5 kb relative to the transcription start site). While the −1.5 kb promoter retained transcriptional activity comparable to the full-length (−2 kb) control, further truncation to −1 kb or −0.5 kb resulted in a significant loss of activation. **G** Western blot after 72 h of gemcitabine treatment shows that Reptin-knockout reduces mitophagy protein levels; this effect is reversed by rescue with WT Reptin but not with K456R. **H** Mtphagy staining (confocal microscopy) after 72 h of gemcitabine treatment. Reptin-knockout attenuates mitophagy; this effect is reversed by rescue with WT Reptin but not with K456R. Scale bar: 25 μm. **I** Electron microscopy after gemcitabine treatment (72 h): Reptin-knockout reduces mitophagolysosomes; this effect is reversed by rescue with WT Reptin but not with K456R. Scale bar: 500 nm. **J** Flow cytometric analysis of mitophagy using the mt-Keima reporter. The percentage of cells with low green fluorescence and high red fluorescence (indicative of mitophagic mitochondria in lysosomes) was quantified. Reptin-knockdown reduced this population, an effect reversed by reconstitution with WT Reptin, but not with the K456R mutant. Data represent the mean ± SEM (*n* = 3 independent experiments). One-way ANOVA (**C**, **D**, **E**, **F**, **H**, **I**, **J**) was used.

To explore the downstream pathway, we performed Reptin chromatin immunoprecipitation sequencing (ChIP-seq) in NOZ cells (2 biological replicates). Enrichment analysis of the 33,827 associated genes identified significant enrichment for the mitophagy pathway (ranking highly in both GO and KEGG analyses; Fig. 5A, B). Notably, 6 significant peaks were detected in the transcription start site (TSS) region of PINK1, a key mitophagy gene (Supplementary Fig. 3B). Mitophagy primarily relies on either the ubiquitin-dependent pathway initiated by PINK1 or the ubiquitin-independent pathway mediated by BCL2 Interacting Protein 3 (BNIP3), BCL2 Interacting Protein 3 Like (BNIP3L), or FUN14 Domain Containing 1 (FUNDC1) [16]. RT-qPCR validation showed that, following gemcitabine treatment, either SENP3-overexpression or Reptin-knockdown specifically reduced PINK1 mRNA levels, while the expression of BNIP3, BNIP3L, and FUNDC1 remained unchanged (Fig. 5C, Supplementary Fig. 3C, D). Reconstitution with WT Reptin restored PINK1 expression, whereas the K456R mutant did not (Fig. 5C). When antioxidant NAC was applied to maintain constant ROS levels, overexpression of SENP3 still led to a decrease in PINK1 mRNA, indicating that SENP3 regulates PINK1 independently of ROS (Supplementary Fig. 3E). ChIP-qPCR confirmed that knockdown of Reptin reduced the enrichment score at the PINK1 promoter, while reconstitution with WT Reptin increased this enrichment, an effect not observed with the K456R mutant (Fig. 5D, Supplementary Fig. 3F). Dual-luciferase reporter assays (using a pGL3 vector containing the PINK1 promoter region [-2 kb]) confirmed that overexpression of WT Reptin activated PINK1 transcription, while the K456R mutant lacked this function (Fig. 5E). We further generated truncated variants of the PINK1 promoter (1.5 kb, 1 kb, and 0.5 kb in length) and performed dual-luciferase reporter assays. The results showed that, compared to the 2 kb promoter, the 1.5 kb variant exhibited no significant difference in transcriptional activation, while the 1 kb and 0.5 kb truncations led to a marked decrease in the ability to activate PINK1 transcription (Fig. 5F). These results pinpoint the critical Reptin-responsive element to the region between −1.5 kb and −1 kb upstream of the transcription start site. Collectively, these findings demonstrate that SUMOylated Reptin transcriptionally upregulates PINK1.

Further experiments revealed that SUMOylated Reptin regulates mitophagy. Following gemcitabine treatment, either SENP3-overexpression or Reptin-knockdown reduced the levels of mitophagy-related proteins (Fig. 5G, Supplementary Fig. 3G−I). Both Mtphagy dye staining combined with confocal microscopy (Fig. 5H) and electron microscopy for direct observation of autophagolysosomes (Fig. 5I), as well as flow cytometric analysis using the mt-Keima mitophagy reporter (Fig. 5J), confirmed that Reptin-knockdown attenuated mitophagy, an effect reversed by reconstitution with WT Reptin but not with the K456R mutant. Thus, SUMOylated Reptin induces mitophagy.

**Fig. 6:**
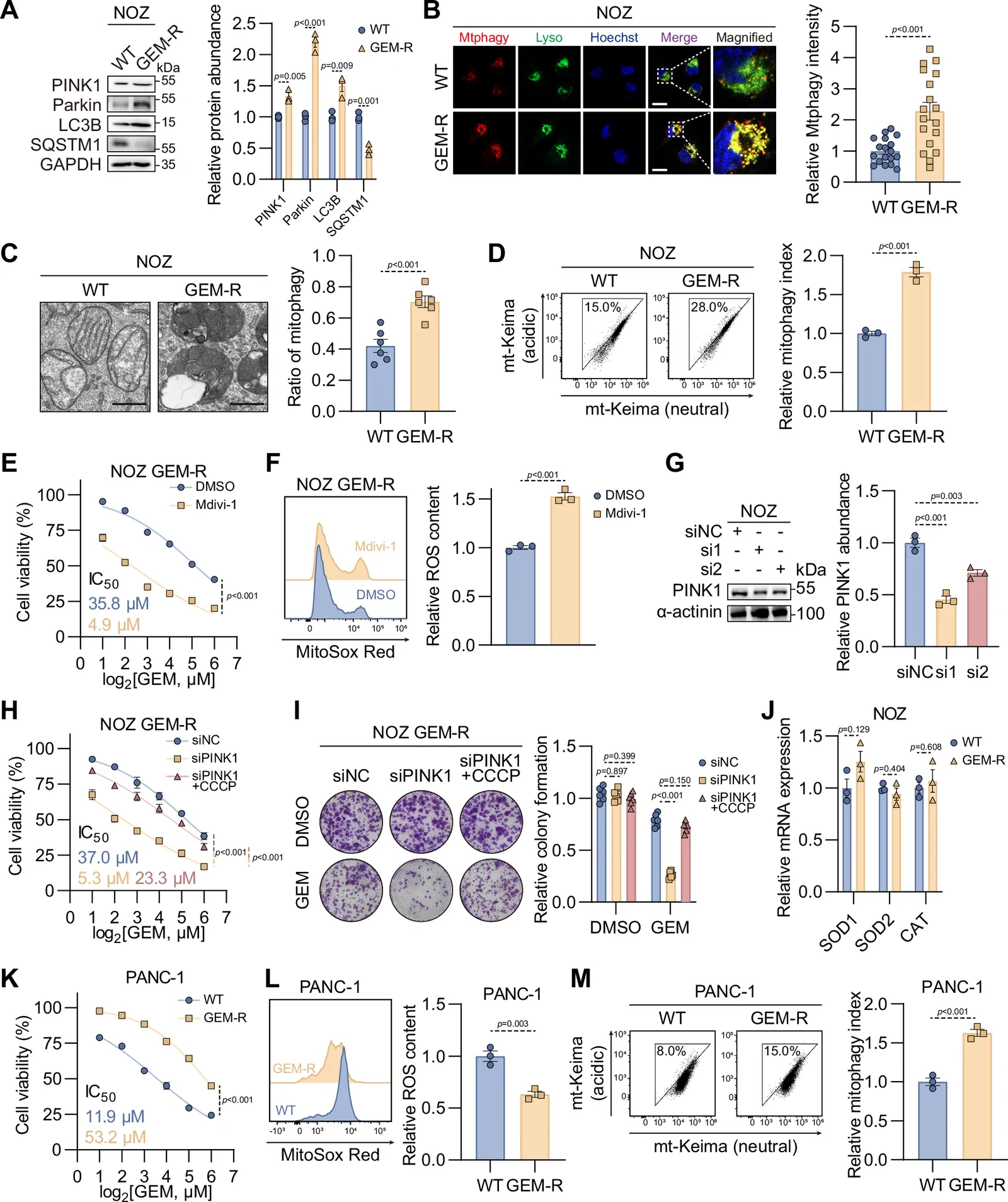
Mitophagy promotes gemcitabine resistance in gallbladder and pancreatic cancer cells. **A**–**C** Western blot (**A**), Mtphagy staining (**B**), electron microscopy (**C**), and mt-Keima reporter assays (**D**) after treatment with 4 μM gemcitabine for 72 h: mitophagy levels in gemcitabine-resistant GBC cell line (GEM-R) are significantly higher than in wild-type (WT) NOZ cells. Scale bars: (**B**) 25 μm, (**C**) 500 nm. **E** Cell viability assays: the mitophagy inhibitor Mdivi-1 (50 μM) significantly reduces the half-maximal inhibitory concentration (IC50) of gemcitabine in GEM-R cells. **F** Flow cytometry detection of ROS (MitoSox Red staining): after treatment with 4 μM gemcitabine for 72 h, Mdivi-1 (50 μM) significantly increases ROS levels in GEM-R cells. **G** Western blot confirming PINK1 knockdown in GEM-R cells. **H**, **I** Cell viability assays **H** and colony formation assays (**I**) show that PINK1 knockdown reduces gemcitabine resistance in GEM-R cells, an effect reversed by the mitochondrial uncoupler CCCP (10 μM). **J** RT-qPCR analysis shows no significant difference in the expression of antioxidant enzymes (SOD1, SOD2, CAT) between GEM-R and WT cells. **K**–**M** The pro-resistance role of mitophagy is conserved in pancreatic cancer. In gemcitabine-resistant PANC-1 cells, cell viability assays (**K**) confirm increased resistance to gemcitabine; Flow cytometry (MitoSOX Red staining) (**L**) shows lower mitochondrial ROS levels after drug treatment; Flow cytometric analysis using the mt-Keima reporter (**M**) demonstrates enhanced mitophagic activity. Data represent the mean ± SEM (*n* = 3 independent experiments). Student’s t-tests (**A**–**D**, **F**, **J**, **L**, **M**), one-way ANOVA (**I**, **G**), and two-way ANOVA (**E**, **H**, **K**) were was used.

To establish the relationship between mitophagy and gemcitabine resistance, we compared mitophagy levels between GEM-R and WT NOZ cells. Consistent results from Western blot, Mtphagy staining, electron microscopy, and flow cytometric analysis using the mt-Keima mitophagy reporter showed that, at the same gemcitabine concentration, mitophagy levels in GEM-R cells were significantly higher than in WT NOZ cells (Fig. 6A–D). Treatment with the mitophagy inhibitor Mdivi-1 [25] reversed the resistance of GEM-R cells (Fig. 6E) and increased intracellular ROS levels (Fig. 6F). We knocked down PINK1 in GEM-R cells using two distinct siRNAs. The siRNA with higher knockdown efficiency (si1, Fig. 6G) was selected for subsequent functional assays. CCK-8 and colony formation assays confirmed that PINK1 knockdown reduced the gemcitabine resistance of GEM-R cells, an effect that could be reversed by treatment with the mitochondrial uncoupler CCCP (Fig. 6H, I). Additionally, the expression levels of antioxidant enzymes (Superoxide Dismutase 1 [SOD1], Superoxide Dismutase 2 [SOD2], and Catalase [CAT]) showed no significant difference between GEM-R and WT cells (Fig. 6J). To validate the generalizability of these findings, we assessed drug resistance, ROS levels, and mitophagic activity in gemcitabine-resistant derivatives of pancreatic cancer PANC-1 cells (Fig. 6K–M). We confirmed that these resistant cells similarly exhibited lower ROS levels and enhanced mitophagic capacity after drug treatment. These results indicate that mitophagy is a key mechanism by which gemcitabine-resistant cancer cells maintain a low ROS state.

### The Reptin inhibitor CB-6644 enhances gemcitabine sensitivity in GBC by suppressing mitophagy

**Fig. 7:**
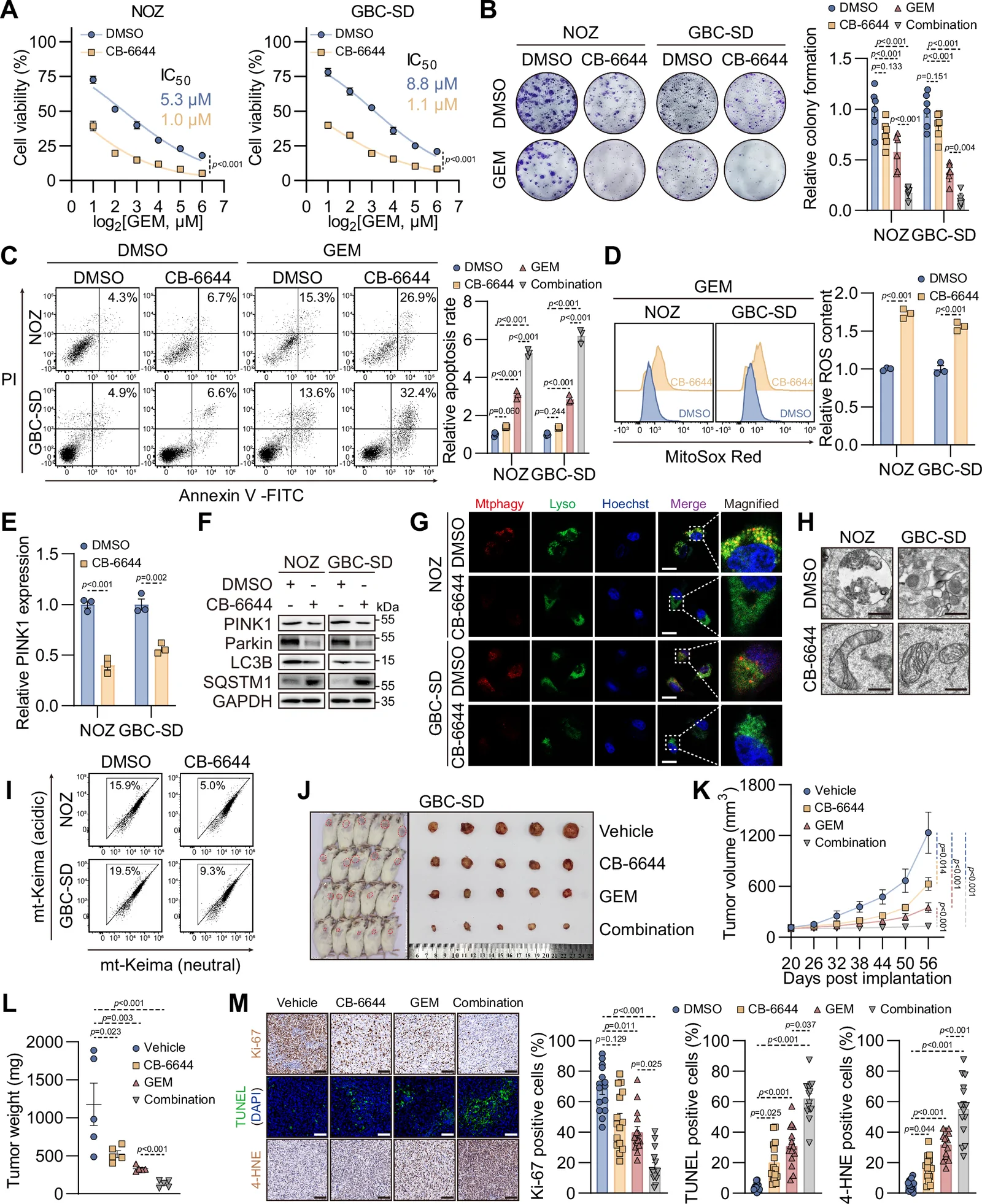
The Reptin inhibitor CB-6644 enhances gemcitabine sensitivity in GBC. **A** Cell viability assays: 200 nM CB-6644 significantly reduces the half-maximal inhibitory concentration (IC50) of gemcitabine in NOZ/GBC-SD cells after 72 h of gemcitabine treatment. **B** Colony formation assays: 200 nM CB-6644 significantly reduces the number of colonies after 72 h of gemcitabine treatment. **C** Apoptosis detection (Annexin V-FITC/PI): 200 nM CB-6644 significantly increases the apoptosis rate after 72 h of gemcitabine treatment. **D** Flow cytometry detection of ROS (H2DCFDA staining): 200 nM CB-6644 significantly increases intracellular ROS levels after 72 h of gemcitabine treatment. **E** RT-qPCR analysis: 200 nM CB-6644 significantly inhibits PINK1 mRNA expression after 72 h of gemcitabine treatment. **F** Western blot: 200 nM CB-6644 significantly reduces levels of mitophagy proteins after 72 h of gemcitabine treatment. **G** Mtphagy staining: 200 nM CB-6644 significantly attenuates mitophagy after 72 h of gemcitabine treatment. Scale bar: 25 μm. **H** Electron microscopy analysis: 200 nM CB-6644 significantly reduces mitophagolysosomes after 72 h of gemcitabine treatment. Scale bar: 500 nm. **I** Flow cytometric analysis using the mt-Keima reporter: 200 nM CB-6644 significantly attenuates mitophagy after 72 h of gemcitabine treatment. **J** Gross photographs of the xenograft tumor experiment in NOD-Scid mice. **K** Tumor growth curves in each treatment group. **L**, **M** Tumor weight (**L**) and staining analysis (**M**) at the endpoint. In the combination treatment group, compared with the gemcitabine monotherapy group, tumors exhibit significantly reduced weight, reduced proliferation index (Ki-67), increased apoptosis rate (TUNEL), and enhanced oxidative damage (4-HNE). Scale bar: 100 μm. Data represent the mean ± SEM (*n* = 3 independent experiments in (**A**, **C**, **D**, **E**, **G**, **H**, **I**); *n* = 6 independent experiments in (**B**); *n* = 5 animals per group in (**J**–**M**). Two-way ANOVA (**A**, **K**), one-way ANOVA (**B**, **C**, **L**, **M**[4-HNE]), Student’s t-tests (**D**, **E**), and Kruskal-Wallis test (L[Ki-67 and TUNEL]) were used.

The Reptin-specific inhibitor CB-6644 exerts antitumor effects by inhibiting its ATPase activity [26]. To evaluate the impact of CB-6644 on gemcitabine sensitivity, we treated GBC cells with CB-6644 combined with gemcitabine. Cell viability assays, colony formation assays, and apoptosis analyses demonstrated that CB-6644 significantly enhanced gemcitabine-induced cytotoxicity and promoted apoptosis (Fig. 7A–C). Mechanistically, ROS levels in the combination group were significantly higher than those in the gemcitabine monotherapy group (Fig. 7D). Additionally, RT-qPCR showed that CB-6644 inhibited PINK1 transcription (Fig. 7E), while Western blot, Mtphagy staining, electron microscopy, and mt-Keima mitophagy reporter analysis revealed that CB-6644 suppressed mitophagy (Fig. 7F–I, Supplementary Fig. 4A–D), indicating that the transcriptional activation of PINK1 by SUMOylated Reptin is dependent on its ATPase activity. To confirm the specificity of CB-6644 toward SUMOylated Reptin, cell viability assays demonstrated that CB-6644 resensitized cells reconstituted with WT Reptin following Reptin knockout, but not those reconstituted with the K456R mutant (Supplementary Fig. 4E). This indicates that CB-6644 specifically inhibits the ATPase activity of SUMOylated Reptin, likely by targeting its nuclear-localized form.

To systematically assess the role of CB-6644 in gemcitabine sensitivity of GBC, we established a NOD-Scid mouse xenograft model (Supplementary Fig. 4F). GBC-SD cells were subcutaneously inoculated into the right flank of mice, and after tumor formation, animals were randomly assigned to receive intraperitoneal injections of saline, CB-6644 monotherapy, gemcitabine monotherapy, or combination therapy. Dynamic monitoring showed that tumor volume and weight in the combination group were significantly smaller than those in the gemcitabine monotherapy group (Fig. 7J-L). Further analyses using Ki-67, TUNEL, and 4-HNE staining revealed that, compared with the gemcitabine monotherapy group, the combination group exhibited a significantly reduced proliferation index, a significantly increased apoptosis rate, and significantly enhanced oxidative damage (Fig. 7M). We further measured plasma concentrations of CB-6644 after intraperitoneal injection in mice and found that it was rapidly cleared from circulation within 24 h (Supplementary Fig. 4G). Tissue distribution analysis revealed that CB-6644 concentrations were higher in healthy tissues (notably in the liver and spleen) than in tumor tissue (Supplementary Fig. 4H). In addition, H&E staining of the liver and kidney did not reveal any apparent damage in these organs (Supplementary Fig. 4I), and serum levels of alanine aminotransferase (ALT) and aspartate aminotransferase (AST) showed no significant elevation (Supplementary Fig. 4J), suggesting a favorable safety profile of CB-6644.

Taken together, these findings demonstrate that CB-6644 enhances gemcitabine sensitivity in GBC both in vitro and in vivo by inhibiting Reptin ATPase activity, blocking the PINK1-mediated mitophagy pathway, and increasing ROS levels.

### SENP3 and Reptin are associated with gemcitabine response in GBC patients

**Fig. 8:**
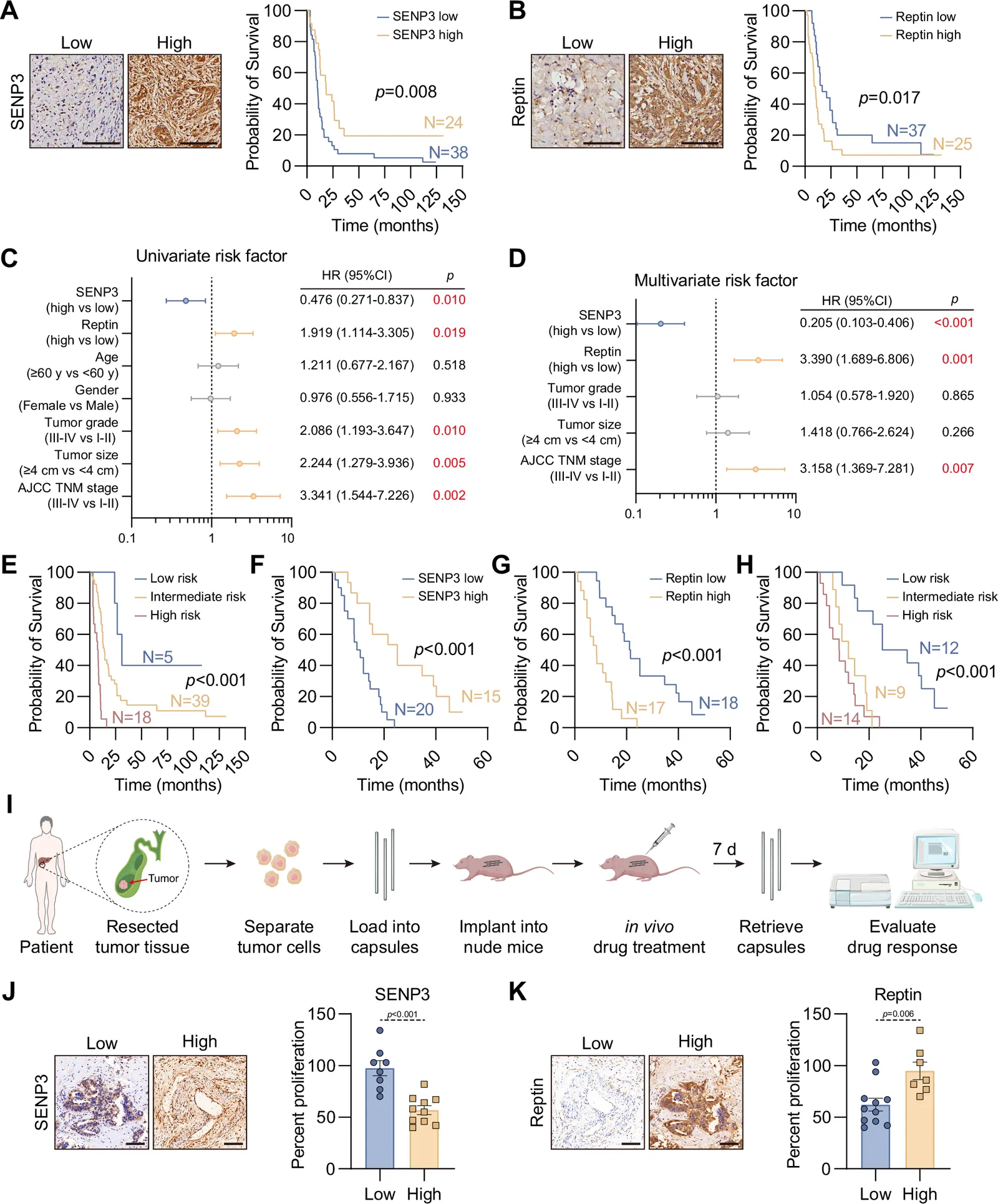
Low SENP3 expression and high Reptin expression predict gemcitabine resistance and poor prognosis in GBC. **A**, **B** GBC patients were stratified based on SENP3 (**A**) and Reptin (**B**) expression levels in formalin-fixed paraffin-embedded specimens. Kaplan-Meier analysis was used to evaluate overall survival of GBC patients who received postoperative gemcitabine treatment (Cohort 1). Scale bar: 100 μm. **C**, **D** Univariate (**C**) and multivariate (**D**) Cox regression analyses of patients in Cohort 1. **E** Patients in Cohort 1 were stratified based on SENP3 and Reptin expression levels: high-risk group (low SENP3 + high Reptin), intermediate-risk group (low SENP3 + low Reptin or high SENP3 + high Reptin), and low-risk group (high SENP3 + low Reptin). Kaplan-Meier analysis was used to evaluate overall survival in each group. **F**–**H** Validation of the prognostic value of SENP3, Reptin, and the combined risk model in an independent cohort. Kaplan-Meier survival analyses in Cohort 2 (*n* = 35) confirm that high SENP3 expression (**F**), low Reptin expression (**G**), and low-risk stratification (**H**) are each associated with significantly longer overall survival. (**I**) Schematic of the mini-PDX model establishment process. **J**, **K** mini-PDX model (Cohort 3) shows that SENP3 expression (**J**) and Reptin expression (**K**) based on tissue scoring are negatively and positively correlated with the proliferation rate of GBC cells under gemcitabine treatment, respectively. Scale bar: 100 μm. Data represent the mean ± SEM. Student’s t-tests were used in (**G**, **H**).

To investigate the clinical relevance of SENP3 and Reptin with gemcitabine sensitivity and prognosis in GBC patients, we analyzed tissue samples from 62 GBC patients who received postoperative gemcitabine chemotherapy (Cohort 1). Patients with high SENP3 expression had significantly longer OS than those with low expression (Fig. 8A, Supplementary Table 1), whereas patients with high Reptin expression had significantly shorter OS than those with low expression (Fig. 8B, Supplementary Table 1). Univariate Cox regression analysis in Cohort 1 revealed that SENP3 expression, Reptin expression, tumor grade, tumor size, and TNM stage were significantly associated with prognosis (Fig. 8C). Multivariate Cox regression analysis incorporating these factors showed that high SENP3 expression was an independent protective factor, while high Reptin expression and advanced TNM stage were independent risk factors (Fig. 8D). Based on the multivariate analysis, we constructed a prognostic prediction model using SENP3 and Reptin expression. Patients stratified into high-risk (low SENP3 + high Reptin), intermediate-risk (low SENP3 + low Reptin or high SENP3 + high Reptin), and low-risk (high SENP3 + low Reptin) groups exhibited progressively longer OS, with significant differences (Fig. 8E, Supplementary Table 1). Furthermore, to validate these findings, we followed a separate validation cohort of 35 GBC patients (Cohort 2). This independent cohort confirmed that the expression levels of SENP3 and Reptin, as well as the derived risk stratification, consistently stratified patient prognosis (Fig. 8F–H, Supplementary Table 2). These results further validate SENP3 and Reptin as accurate biomarkers for predicting chemotherapy sensitivity in GBC patients.

Additionally, we analyzed gemcitabine sensitivity in 18 GBC mini-patient derived xenograft (mini-PDX) models (Cohort 3) and assessed SENP3 and Reptin expression in corresponding patient paraffin sections. The mini-PDX model involves isolating tumor cells from GBC patients’ tumor tissues, encapsulating them in special capsules, and transplanting these capsules into nude mice. Following systemic drug administration, the capsules are retrieved to assess tumor cell viability and calculate the relative proliferation rate, thereby evaluating the patient’s sensitivity to chemotherapeutic agents [27] (Fig. 8I). Patients with high SENP3 expression showed significantly lower relative proliferation rates than those with low expression (Fig. 8J, Supplementary Table 3), while patients with high Reptin expression had significantly higher relative proliferation rates than those with low expression (Fig. 8K, Supplementary Table 3).

These results indicate that SENP3 and Reptin can serve as important molecular markers for predicting gemcitabine sensitivity in GBC patients, consistent with our cellular and animal experimental findings.

## Discussion

Previous studies reported that gemcitabine eliminates tumor cells by inducing ROS accumulation [28]. However, this study revealed that under gemcitabine stimulation, gemcitabine-resistant GBC cells exhibit significantly lower ROS levels than WT cells, a state maintained by enhanced mitophagy. Mechanistic investigations identified a key mitophagy/ROS/SENP3/Reptin loop: a low ROS environment reduces SENP3 protein levels, relieving its inhibition of deSUMOylation at the K456 site of Reptin. This promotes nuclear localization of SUMOylated Reptin, which transcriptionally activates PINK1, a core mitophagy regulator, thereby enhancing mitophagy to clear excess ROS and maintain a low ROS state. This forms a self-reinforcing cycle sustaining the low ROS condition and gemcitabine-resistant phenotype. Clinical samples and mini-PDX models confirmed that low SENP3 expression and high Reptin expression are significantly associated with poor chemotherapy response in GBC patients. Targeted intervention at key nodes of this loop (e.g., inhibition of Reptin with CB-6644) effectively reverses gemcitabine resistance. These findings reveal the core mechanism underlying gemcitabine resistance in GBC, providing a theoretical basis for developing SENP3/Reptin-based efficacy prediction models and novel combination therapies targeting this pathway.

This study uncovered a novel gemcitabine resistance mechanism wherein a low ROS environment drives mitophagy: in resistant GBC cells, the mitophagy/ROS/SENP3/Reptin loop actively maintains and enhances mitophagic capacity under low ROS, forming an adaptive mechanism preventing ROS elevation. Tumor cells exhibit higher intracellular ROS levels than normal cells, which is a critical basis for the selective killing by chemotherapeutic agents [29]. Thus, high intracellular ROS levels in tumor cells are pivotal for chemosensitivity. Drug-resistant tumor cells typically counter oxidative damage through multiple mechanisms [11], including metabolic reprogramming (e.g., enhanced Warburg effect) to reduce ROS production [30] and upregulation of endogenous antioxidant systems (e.g., glutathione synthetase, superoxide dismutase [SOD], catalase [CAT]) [31] to scavenge excess ROS. As a ROS-scavenging mechanism, mitophagy has gained increasing attention in tumor drug resistance [12]. Notably, mitophagy is a form of selective macroautophagy, distinct from classical non-selective macroautophagy (often simply referred to as “autophagy”) in substrate recognition, degradation mechanisms, and functional regulation [32]. The classical mitophagy pathway involves chemotherapy-induced high ROS causing mitochondrial damage, which activates either the PINK1/Parkin-mediated ubiquitin-dependent pathway or receptor-mediated (BNIP3, BNIP3L, FUNDC1) ubiquitin-independent pathways to clear damaged mitochondria, thereby reducing intracellular ROS levels, inhibiting drug-induced oxidative damage and apoptosis, and leading to chemoresistance [17]. This mechanism plays important roles in chemoresistance in small-cell lung cancer [33], non-small-cell lung cancer (NSCLC) [34], cervical cancer [35] and pancreatic cancer [36]. In this context, the “low-ROS-driven mitophagy” self-reinforcing model identified in this study represents a novel adaptive resistance mechanism. It not only enriches the ROS-mitophagy regulatory framework but also provides a new perspective for understanding resistance in other cancers.

This study achieved key advances in elucidating the molecular mechanisms of the loop. First, regarding SENP3, while previous research focused on genomic stability [37] and immune regulation [38], we demonstrate for the first time that SENP3, as a ROS sensor, directly influences gemcitabine sensitivity in GBC. We identified Reptin as a novel target of SENP3, with SUMOylation at K456 acting as a molecular switch for its nuclear localization and activity. Notably, the K456R mutation blocks SUMOylation without affecting the binding between Reptin and SENP3, suggesting that SUMOylation is not directly involved in their interaction. Second, regarding AAA+ ATPase Reptin, although implicated in pro-cancer processes such as transcriptional regulation [22], its role in tumor drug resistance was largely unknown. A single report suggests that Reptin-knockdown enhances cisplatin sensitivity in NSCLC, but the mechanism remains unclear [39]. This study fills the gap in understanding the role of Reptin in GBC and is the first to clarify that Reptin transcriptionally activates PINK1, initiating the PINK1/Parkin-mediated mitophagy pathway. Third, we found that SENP3 and Reptin expression levels are closely associated with gemcitabine sensitivity in GBC patients, supporting their use in a prediction model. Finally, we confirmed that the pro-resistance function of Reptin depends on its ATPase activity [40, 41]. CB-6644, a specific inhibitor of this activity, blocks PINK1 transcriptional activation and reverses resistance. Its efficacy depends on the SUMOylated state of Reptin, likely because SUMOylation is required for Reptin’s nuclear translocation where CB-6644 exerts its effect, providing direct evidence for targeted therapy.

In summary, this study elucidated a novel mechanism of gemcitabine resistance in GBC, wherein low ROS activates mitophagy through a positive feedback loop involving mitophagy/ROS/SENP3/Reptin that sustains low ROS levels via enhanced mitophagy. Investigating this mechanism not only deepens our understanding of adaptive tumor resistance but also identifies new biomarkers (SENP3/Reptin) and targeted therapeutic agents (e.g., CB-6644). Blocking this loop holds promise for improving gemcitabine sensitivity in GBC patients.

## Methods

### Patients and specimens

Three distinct cohorts of GBC patients were recruited for this study. For the first cohort, a tissue microarray containing 62 cases of GBC tumor tissues, accompanied by comprehensive clinicopathological and follow-up data, was retrospectively obtained from GBC patients who underwent radical cholecystectomy before receiving gemcitabine-based chemotherapy at Renji Hospital affiliated with Shanghai Jiao Tong University School of Medicine between January 2008 and April 2015, with the patients’ consent. For the second cohort, 35 cases of GBC tumor tissues, accompanied by comprehensive clinicopathological and follow-up data, was retrospectively obtained from GBC patients who underwent radical cholecystectomy before receiving gemcitabine-based chemotherapy at Shanghai Sixth People’s Hospital Affiliated to Shanghai Jiao Tong University School of Medicine between May 2020 and July 2024, with the patients’ consent. The inclusion criteria for patients were: (1) definitive GBC diagnosis by pathology. (2) radical cholecystectomy, including complete resection of primary tumorous tissues with confirmation of negative margins by histological examination. (3) absence of radiotherapy or chemotherapy prior to surgery. (4) receipt of gemcitabine-based chemotherapy post-surgery. The sample size for the Cohort 1 and 2 was determined by the total number of consecutive, eligible patients available during the study period, which is a standard approach for retrospective biomarker studies.

In the third cohort, 18 cases of primary GBC tissues were utilized to assess gemcitabine sensitivity in mini-PDX models, alongside the acquisition of relevant clinical information from patients. These primary tissues were obtained via radical cholecystectomy or tissue biopsy before the initiation of guided chemotherapy, guided by mini-PDX results, at Shanghai Sixth People’s Hospital Affiliated with Shanghai Jiao Tong University School of Medicine and Renji Hospital affiliated with Shanghai Jiao Tong University School of Medicine between July 2016 and August 2023, with the patients’ consent.

This study received approval from the Ethics Committee of Shanghai Sixth People’s Hospital Affiliated with Shanghai Jiao Tong University School of Medicine (No. 2020-243), and written informed consent was obtained from all subjects involved in the study.

### Cell culture and reagents

NOZ cells (CTCC-003-0099) and GBC-SD cells (CTCC-400-0165) were procured from Zhejiang Meisen Cell Technology Co., Ltd (Jinhua, China), while human embryonic kidney 293 T (HEK293T) cells were acquired from the American Type Culture Collection. The wild-type and gemcitabine-resistant PANC-1 cells were purchased from Yaji Biotechnology Co., Ltd. (Shanghai, China). All cell lines were recently authenticated by short tandem repeat (STR) profiling and tested negative for mycoplasma contamination. NOZ cells were cultured in William’s E medium (Gibco), whereas GBC-SD, PANC-1 and 293T cells were maintained in Dulbecco’s Modified Eagle’s Medium (Gibco). Wild-type NOZ cells were subjected to escalating concentrations of gemcitabine (HY-17026, MedChem Express) ranging from 500 nM to a final concentration of 16 μM over a 24-week period and were subsequently cultured in 16 μM gemcitabine to sustain their chemoresistance, leading to the development of GEM-R cells. All cell lines were supplemented with 10% fetal bovine serum (FBS, Gibco) and penicillin/streptomycin and were incubated in a humidified chamber with 5% CO_2_ at 37 °C. H_2_O_2_ (323381, Sigma-Aldrich) and NAC (A7250, Sigma-Aldrich) were employed to modulate intracellular ROS levels, while MitoSOX Red (HY-D1055, MedChem Express) was utilized for intracellular ROS detection via flow cytometry. Mdivi-1 (HY-15886, MedChem Express) was utilized for mitophagy inhibition, CCCP (HY-100941, MedChem Express) was utilized for mitophagy activation, and CB-6644 (HY-114429, MedChem Express) was used to hinder the ATPase activity of Reptin.

### Plasmids, shRNA, and sgRNA

Flag-tagged SENP3 was inserted into the pCDH or pcDNA3.1 vector, while HA-tagged WT or mutant (K115R, K132R, K269R, K288R, K368R, K456R) Reptin was incorporated into the pcDNA3.1 vector. SENP3-targeting shRNAs and a non-specific control shRNA (shNC) were integrated into the pLKO.1 vector. Reptin-targeting sgRNA and a non-specific control sgRNA (sgNC) were inserted into the lentiCRISPR-V2 vector. The 2 kb region upstream of the transcription start site of PINK1 was designated as its promoter region, cloned into the pGL3-basic vector, and labeled as PINK1-Luc.

### Virus production and infection

293 T cells cultured in 10 cm dishes reached optimal transfection readiness at 70% to 80% confluency. Co-transfection was performed using 12 µg of the necessary plasmids (overexpression constructs, shRNAs, or sgRNAs), along with 9 µg of psPAX2 and 3 µg of pMD2.G, employing 50 µl of polyethyleneimine. Following transfection, the 293 T cells were maintained at 37 °C, with the transfection medium being refreshed after 4 h. Virus-containing medium was harvested between 48 to 72 h post-transfection and augmented with 5 µg/ml polybrene for infecting target cells in dishes or microplates, a process lasting 12 to 24 h. Subsequently, the infected cells underwent positive selection with 2.5 µg/ml puromycin to eliminate uninfected cells and establish stable cell lines.

### Generation of single-cell Reptin knockout clones

The modified lentiCRISPR-V2 vector, housing Cas9 nuclease and sgRNA targeting the sixth exon in the Reptin genomic locus, was employed to establish stable Reptin knockout (RKO) cell lines in both NOZ and GBC-SD cells. Subsequently, single-cell knockout clones were generated, and the knockout efficiency was validated through genomic PCR sequencing and immunoblotting.

### Cell viability assays

Cells in single-cell suspension were seeded at a density of 4000 cells per well for chemical reagent treatment or 2000 cells per well for growth rate assays in 96-well plates containing 100 µl of culture medium. Cell viability was evaluated using the Cell Counting Kit-8 (CCK-8) assay at specified time points: 72 h post-chemical reagent treatment or every 24 h after plating. Briefly, 10 µl of CCK-8 solution was directly added to the cells, followed by a 3 h incubation at 37 °C, and absorbance was measured at 450 nm.

### Colony formation assays

For colony formation, GBC cells in single-cell suspension were seeded and cultured in 6-well plates at a density of 500 cells per well or in 12-well plates at a density of 250 cells per well for 10 days. Subsequently, cells were treated with gemcitabine at the concentration of IC_50_ or vehicle for 96 h. Colonies were then fixed with 4% paraformaldehyde and stained with 0.1% crystal violet.

### Cell apoptosis assays

GBC cells were plated in dishes or microplates overnight and treated with gemcitabine or vehicle for the indicated time and dose. Subsequently, all cells were harvested by trypsinization without EDTA and doubly stained with Annexin V-FITC/PI (C1062, Beyotime Biotechnology) and analyzed using flow cytometry.

### Cell migration assays

GBC cells in single-cell suspension were seeded at a density of 10000 cells per well into the upper compartment of a Transwell chamber (Corning). The lower chamber was filled with 500 µl of DMEM supplemented with 20% FBS. After incubating the cells for 24 h, those in the upper chamber were removed with a cotton swab. Migrated cells were then fixed with 4% paraformaldehyde and stained with 0.1% crystal violet.

### Western blot assays

Standard procedures were followed for immunoblotting. Cell lysates were prepared using radioimmunoprecipitation lysis buffer (50 mM Tris-HCl [pH 7.4], 150 mM NaCl, 1% NP-40, 0.1% SDS, 2 µM EDTA) supplemented with proteinase inhibitor and quantified using the BCA Protein Assay Kit (P0012, Beyotime Biotechnology). Twenty micrograms of protein aliquots were electrophoresed through 10% or 12% SDS polyacrylamide gels and transferred to polyvinyl difluoride membranes (Millipore). Membranes were then blocked in 5% skim milk at room temperature for 1 h, followed by overnight incubation with primary antibodies at 4 °C. Horseradish peroxidase-conjugated secondary antibodies were used, and signals were detected using the ECL Kit (Millipore). Antibodies against SENP3 (5591, 1:1000), SUMO2/3 (4971, 1:1000), PINK1 (6946, 1:500), LC3B (3868, 1:1000), α-actinin (6487, 1:1000) were obtained from Cell Signaling Technology; Antibodies against GAPDH (60004-1-Ig, 1:1000), β-Tubulin (10094-1-AP, 1:1000), SQSTM1 (66184-1-Ig, 1:1000) were obtained from Proteintech; Antibodies against Reptin (A1905, 1:1000), HA (AE008, 1:1000), Lamin A/C (A17319, 1:1000) were obtained from ABclonal. Full, uncropped scans of all Western blots are provided in the Supplementary Material.

### Immunoprecipitation (IP) assays

For exogenous co-IP assays, 293 T cells were transfected with plasmids for 72 h. For endogenous co-IP assays, NOZ cells were used without plasmid transfection. Cells were lysed with IP lysis buffer (50 mM Tris-HCl [pH 8.0], 150 mM NaCl, 1 mM EDTA, 0.5% NP-40, and protease inhibitor cocktail), followed by incubation with antibodies at 4 °C overnight. Protein A/G Magnetic Beads (88803, Thermo Fisher Scientific) were employed to capture antibodies. The immunocomplexes were then washed with lysis buffer and subjected to immunoblotting. For SUMOylation assays, cells were transfected with plasmids for 72 h, lysed with denaturing buffer (50 mM Tris-HCl [pH 6.8], 2% SDS, 40 mM DTT, 5% glycerol, 20 mM NEM, and protease inhibitor cocktail) for 30 minutes, and subjected to immunoblotting following IP with SUMO-IP buffer (20 mM Tris-HCl [pH 8.0], 150 mM NaCl). Antibody against SENP3 (5591, 1:100) was obtained from Cell Signaling Technology; Antibodies against Reptin (A1905, 1:100), HA (AE008, 1:100), Flag (AE005, 1:100) were obtained from ABclonal.

### Mass spectrometry

Beads were resuspended in 100 µl of 50 mM NH_4_HCO_3_, reduced with 10 mM DTT at 56 °C for 1 h, alkylated with 50 mM iodoacetamide at room temperature in the dark for 40 min, and subjected to enzymatic digestion at 37 °C overnight. Salt was removed from the sample using a C18 tip, and extracted peptides were lyophilized to near dryness. Experiments were conducted using a Q Exactive Hybrid Quadrupole-Orbitrap Mass Spectrometer coupled with Easy-nLC1200 (Thermo Fisher Scientific). Data were acquired using a data-dependent top20 method, and raw MS files were analyzed and searched against a protein database using MaxQuant (1.6.2.10).

### Data-Independent Acquisition (DIA) Proteomics

There are 3 biological replicates for the samples. Protein extraction was performed using SDT lysis buffer (4% SDS, 100 mM Tris-HCl [pH 7.6]), with protein concentration determined via BCA assay. A total of 15 μg of each protein sample was subjected to SDS-PAGE on a 4–20% gradient gel, stained with Coomassie Blue R-250, and a pooled QC sample was prepared. Filter-aided proteome preparation (FASP) was used for trypsin digestion, followed by C18 desalting. Peptides were reconstituted in 0.1% formic acid with iRT standards and analyzed via DIA-MS using an Astral mass spectrometer coupled to a Vanquish Neo UHPLC system. Parameters included a 380–980 m/z MS1 scan range (240,000 resolution), 299 DIA windows (2 m/z isolation), and HCD collision energy of 25 eV. Data were processed using DIA-NN with trypsin specificity, allowing 1 missed cleavage, carbamidomethylation of cysteine as a fixed modification, and oxidation of methionine and N-terminal acetylation as variable modifications. Protein identifications were filtered at 1% FDR.

### Chromatin immunoprecipitation Sequencing (ChIP-seq)

There are 2 biological replicates for the samples. NOZ cells were cross-linked with formaldehyde, and chromatin was extracted and sheared into fragments of 200–1000 bp. Immunoprecipitation was performed using antibodies against Reptin (ab91462, 1:50, Abcam), and antibody-protein-DNA complexes were captured using Protein A/G beads, washed, and eluted. DNA was purified and subjected to library preparation, followed by high-throughput sequencing. Data analysis included aligning reads to the reference genome, identifying enriched regions, and annotating peaks.

### Chromatin immunoprecipitation quantitative PCR (ChIP-qPCR)

Following the ChIP procedure described above, the immunoprecipitated DNA was analyzed by quantitative PCR. Specific primers were designed to amplify the promoter region of the PINK1 gene. The relative enrichment of target DNA fragments was calculated by normalizing the amount of immunoprecipitated DNA to the corresponding input DNA, with technical replicates performed for each sample.

### Real-time quantitative PCR (RT-qPCR) assays

Total RNA was extracted from cells using TRIzol Reagent (Invitrogen), and 500 ng of total RNA was reverse transcribed into cDNA using the ABScript Neo RT Master Mix (ABclonal). RT-qPCR was performed in triplicate using the QuantStudio 5 System (Applied Biosystems). Ct values were compared using the 2^−ΔΔCt^ method, with 18S serving as an internal reference gene. Primers used for RT-qPCR are listed in Supplementary Materials.

### Immunofluorescence (IF) assays

GBC cells were seeded into confocal dishes, fixed with 4% paraformaldehyde, permeabilized with 0.1% Triton X-100, and blocked with 5% BSA. Primary antibody against Reptin (A1905, 1:50, ABclonal) was applied overnight at 4 °C, followed by incubation with fluorophore-conjugated secondary antibodies (8889, 1:500, Cell Signaling Technology) and nuclear staining with DAPI dye. Images were captured using a confocal laser scanning microscope.

### Luciferase assays

For luciferase assays, 293 T cells were seeded in 12-well plates at a density of 20000 cells per well and incubated overnight. Transfection of PINK1-Luc and pRL-TK plasmids was performed using polyethyleneimine. Cells were harvested 48 h post-transfection, lysed, and Rluc and Fluc activities were measured using the Dual-Luciferase Reporter Assay System (Promega). Relative luciferase activity was calculated as the ratio of Fluc to Rluc.

### Mitophagy staining

Mitophagy in live cells was monitored using the Mitophagy detection kit MD01 (Dojindo) according to the manufacturer’s instructions. Gemcitabine treatment induced mitophagy, and the level of mitophagy was evaluated based on the Mtphagy dye area of each cell. Colocalization of Mtphagy and lysosomal dyes was also analyzed.

### Mt-Keima mitophagy assay

Cells were transiently transfected with the pHAGE-mt-mKeima plasmid using an appropriate transfection reagent. After transfection, cells were treated with gemcitabine or vehicle for the indicated time and dose. Subsequently, cells were harvested and subjected to dual-excitation flow cytometry (excitation: 488 nm for neutral pH, 561 nm for acidic pH) to quantify the red (lysosomal) and green (mitochondrial) fluorescence signals, reflecting mitochondrial delivery to lysosomes.

### Transmission electron microscopy (TEM) analysis

For TEM, fresh 1 mm^3^ tissues were fixed in Servicebio fixative (G1102) within 1–3 min, then post-fixed with 1% osmium tetroxide. Dehydrated in ethanol/acetone, infiltrated with 812 embedding medium (SPI), polymerized at 60 °C for 48 h. 60–80 nm sections (Leica UC7) on 150-mesh grids were stained with uranyl acetate and lead nitrate, observed via HT7800 TEM (Hitachi).

### Xenograft model

For SENP3-related experiments, 4-week-old male BALB/c nude mice were housed under specific pathogen-free conditions and injected subcutaneously with 2 × 10^6^ NOZ or GBC-SD cells in 100 µl of PBS. Gemcitabine (20 mg/kg) or vehicle (saline) injections were administered intraperitoneally every 3 days, with six mice per group.

For CB-6644-related experiments, 4-week-old male NOD-Scid mice were housed under specific pathogen-free conditions and injected subcutaneously with 2 × 10^6^ GBC-SD cells in 100 µl of PBS. Vehicle (saline), gemcitabine (20 mg/kg), CB-6644 (20 mg/kg), or combination (20 mg/kg gemcitabine and 20 mg/kg CB-6644) injections were administered intraperitoneally every 3 days, with five mice per group.

After tumor formation, mice were randomly allocated into groups. Tumor measurements were taken every 6 days before each injection, and tumor volume was calculated using the formula (length × width^2^)/2. Mice were euthanized at the end of the study, and tumors were collected and weighed. Animal procedures were conducted in accordance with the National Institutes of Health Guidelines and approved by the Institutional Animal Care and Use Committee of Shanghai Sixth People’s Hospital Affiliated to Shanghai Jiao Tong University School of Medicine (No. 2024-0273). The sample size (*n* ≥ 5 mice per group) was chosen based on common practice in similar xenograft studies in our field and was constrained by ethical principles of the 3Rs (Replacement, Reduction, Refinement) to use the minimum number of animals necessary to achieve reliable results.

### Mini-patient derived xenograft (mini-PDX) model

Drug sensitivity was evaluated using the OncoVee mini-PDX assay (LIDE Biotech Inc., China) [27]. GBC tissues were obtained after surgical resection, processed, and implanted subcutaneously in BALB/c nude mice. Gemcitabine (60 mg/kg) or placebo (saline) treatments were administered for 7 days, followed by evaluation of anti-tumor activity based on relative fluorescence units (RFU) using the CellTiter-Glo Luminescent Cell Viability Assay (Promega). Proliferation rates were calculated based on RFU measurements. All procedures were performed under sterile conditions and approved by the Institutional Animal Care and Use Committee.

### Immunohistochemistry (IHC) analysis

Tissue slides were deparaffinized, treated with 3% H_2_O_2_, autoclaved, and incubated with primary antibodies overnight at 4 °C. Biotinylated secondary antibodies were applied, followed by signal amplification and detection using the DAB system. Stained sections were photographed under a light microscope, and protein expression was evaluated based on staining intensity and proportion scores. Antibodies against SENP3 (ab247139, 1:200), Reptin (ab91462, 1:200), and Ki-67 (ab15580, 1:200) were obtained from Abcam. Antibody against 4-HNE (bs-6313R, 1:200) was obtained from Bioss. TUNEL Assay Kit (ab66108) was obtained from Abcam.

### Hematoxylin and eosin (H&E) staining

Tissue sections of liver and kidney were deparaffinized in xylene and rehydrated through a graded series of ethanol to distilled water. The sections were then stained with hematoxylin for nucleus visualization, followed by differentiation in acid alcohol and bluing in ammonia water. Subsequently, the cytoplasm was counterstained with eosin. After staining, the sections were dehydrated through an ascending ethanol series, cleared in xylene, and mounted with a neutral resinous medium. Stained sections were examined and photographed under a light microscope to assess tissue morphology and pathological changes.

### Plasma concentration measurement of CB-6644

Mice received a single intraperitoneal injection of CB-6644. Blood samples were collected at 0.25, 0.5, 1, 2, 4, 8, 12, and 24 h post-injection. Plasma was separated by centrifugation, and the concentration of CB-6644 was quantified using mass spectrometry. The plasma concentration-time profile was analyzed to evaluate the systemic exposure of CB-6644.

### Measurement of serum ALT and AST levels

To assess liver function, the enzymatic activities of ALT and AST in mouse serum were quantified using commercial assay kits (Nanjing Jiancheng Bioengineering Institute, China. Catalog No. Z002-1-1) following the manufacturer’s protocols.

### Statistical analysis

Data were presented as means ± SEM. Statistical analysis was performed using R (4.5.1) software. Kolmogorov-Smirnov test and Shapiro-Wilk test were used for normality tests. For normally distributed quantitative data, Student’s t-tests and analysis of variance (ANOVA) were used; for skewed distributed quantitative data, Mann-Whitney U test and Kruskal-Wallis test were used. Survival analysis was conducted using the Kaplan-Meier method and log-rank test. Cox regression analysis was used for multivariate analysis, and Pearson correlation coefficient was calculated for IHC staining. A two-sided *p-*value < 0.05 was considered statistically significant.

## Supplementary information

Supplementary Tables

Supplementary Figure 1

Supplementary Figure 2

Supplementary Figure 3

Supplementary Figure 4

Supplementary figure legends

Original uncropped images of all blots

Supporting data values

aj-checklist

## Data Availability

All data supporting the findings of this study are available from the corresponding author on reasonable request. The raw sequencing data (ChIP-seq) has been deposited in a public repository (Gene Expression Omnibus, GEO) under the accession code GSE317548.
