# Supplementary material for: SUMOylated Reptin maintains low intracellular ROS levels by activating mitophagy to drive gemcitabine resistance in gallbladder cancer
Source: Cell Death Dis. 2026 Sep 10;17(1):394. doi: 10.1038/s41419-026-08598-0 (PMC13558568; doi:10.1038/s41419-026-08598-0)

Fig. 2C
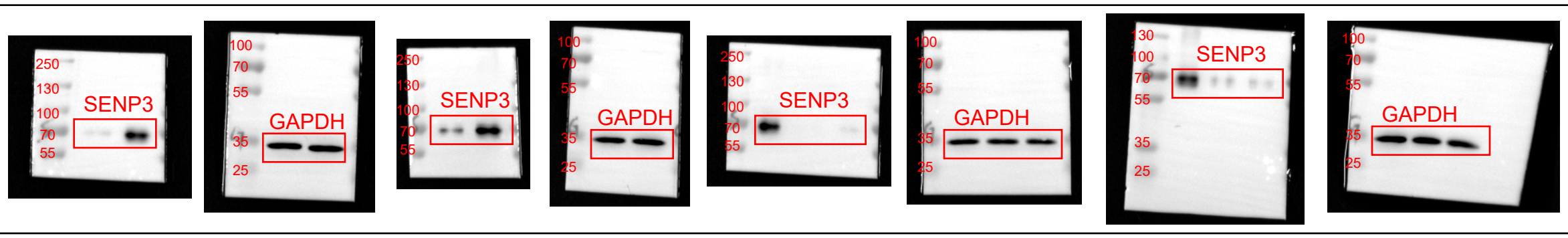

Fig. 4A
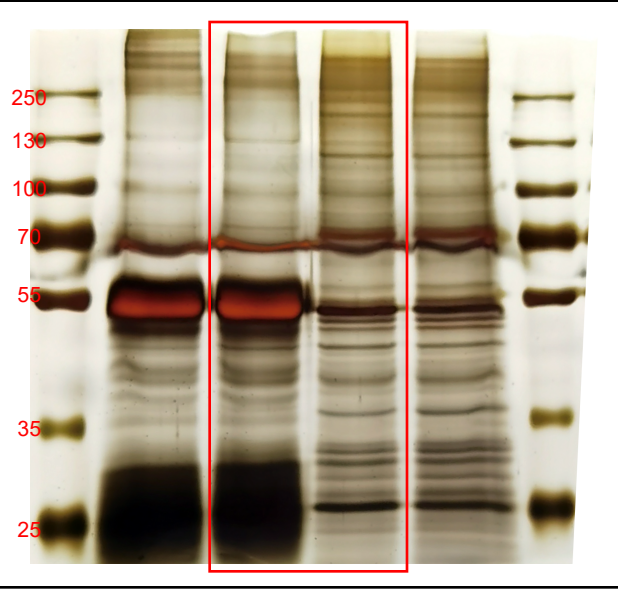

Fig. 4C
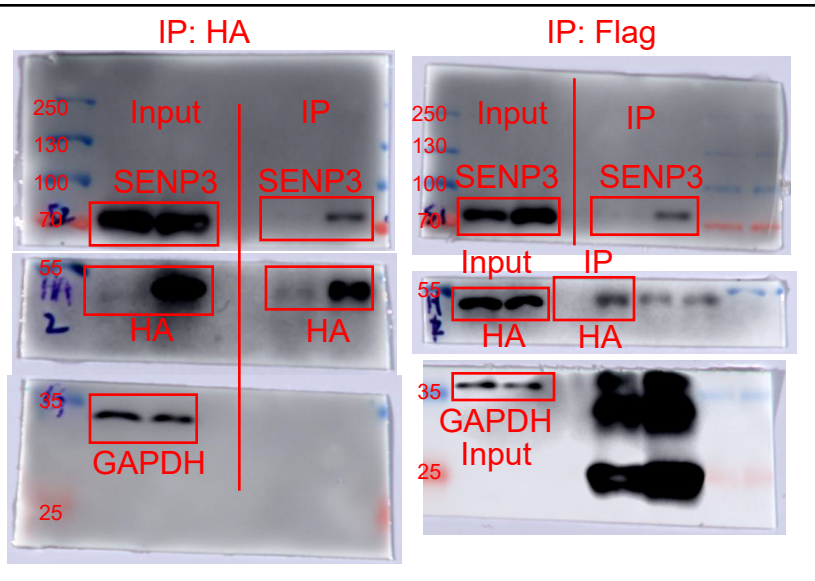

Fig. 4D
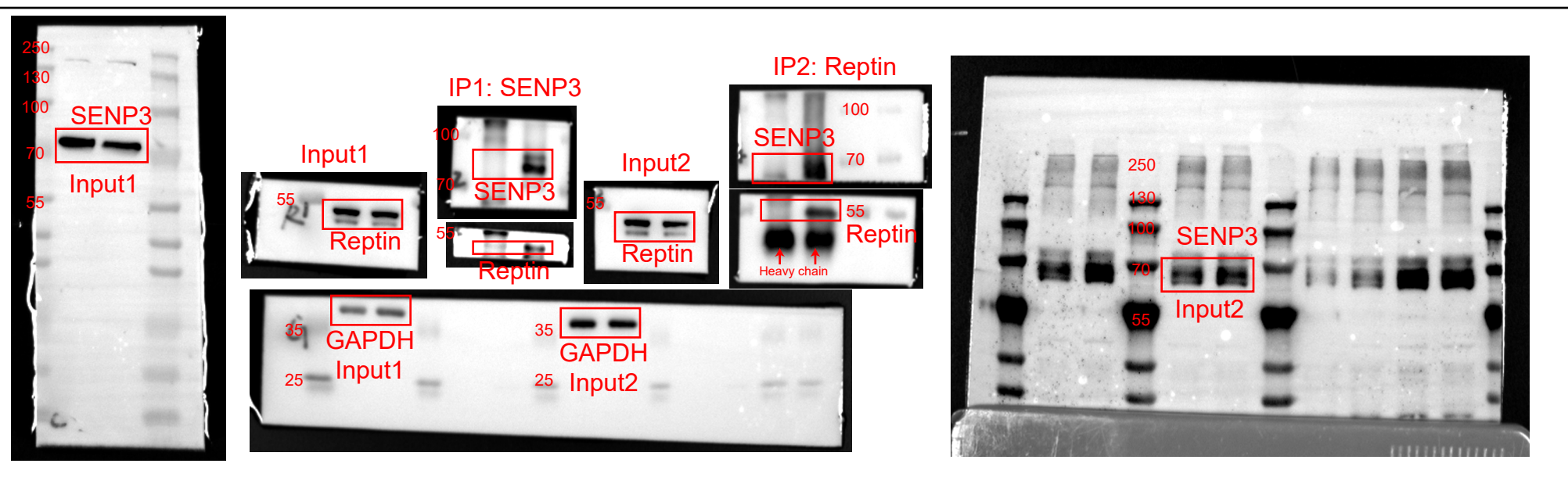

Fig. 4E
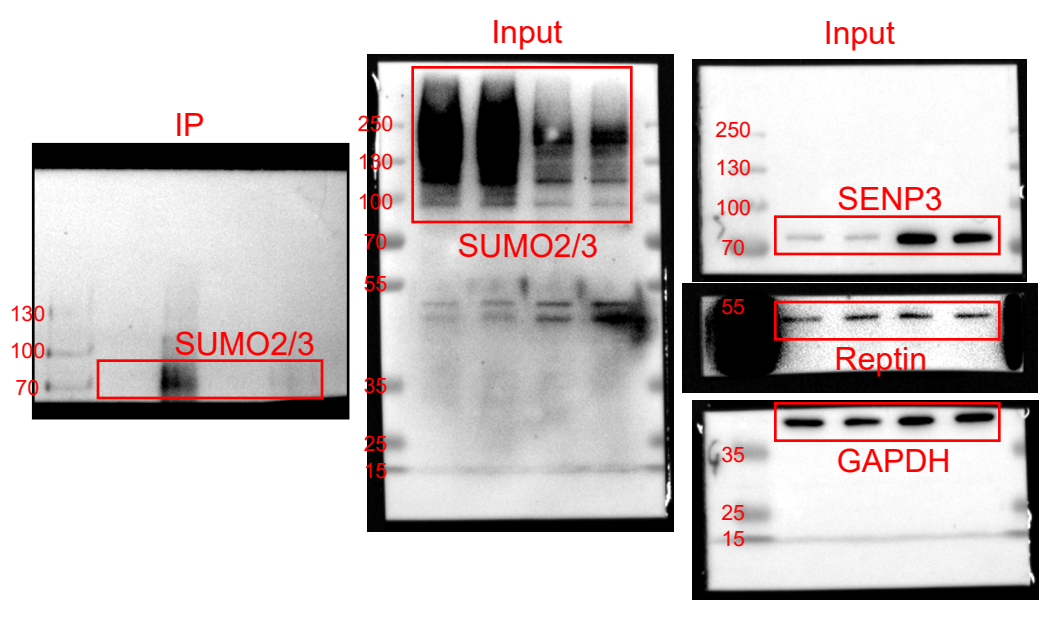

Fig. 4F
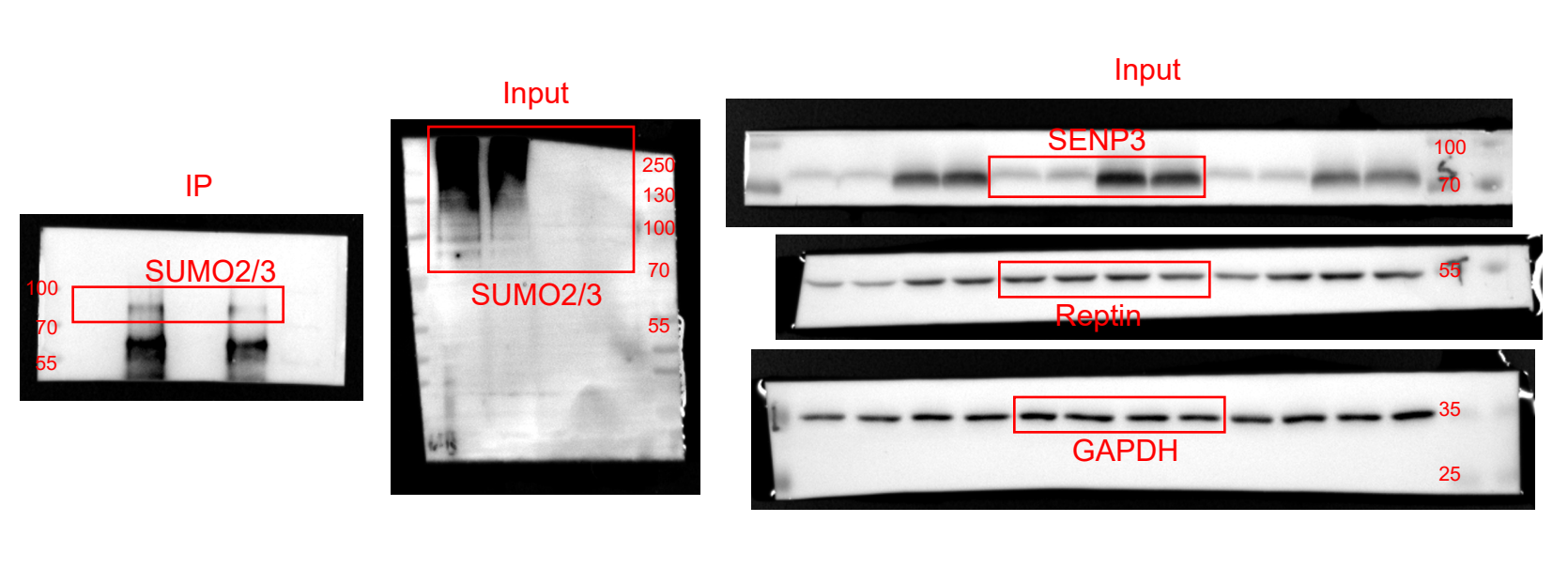

Fig. 4G
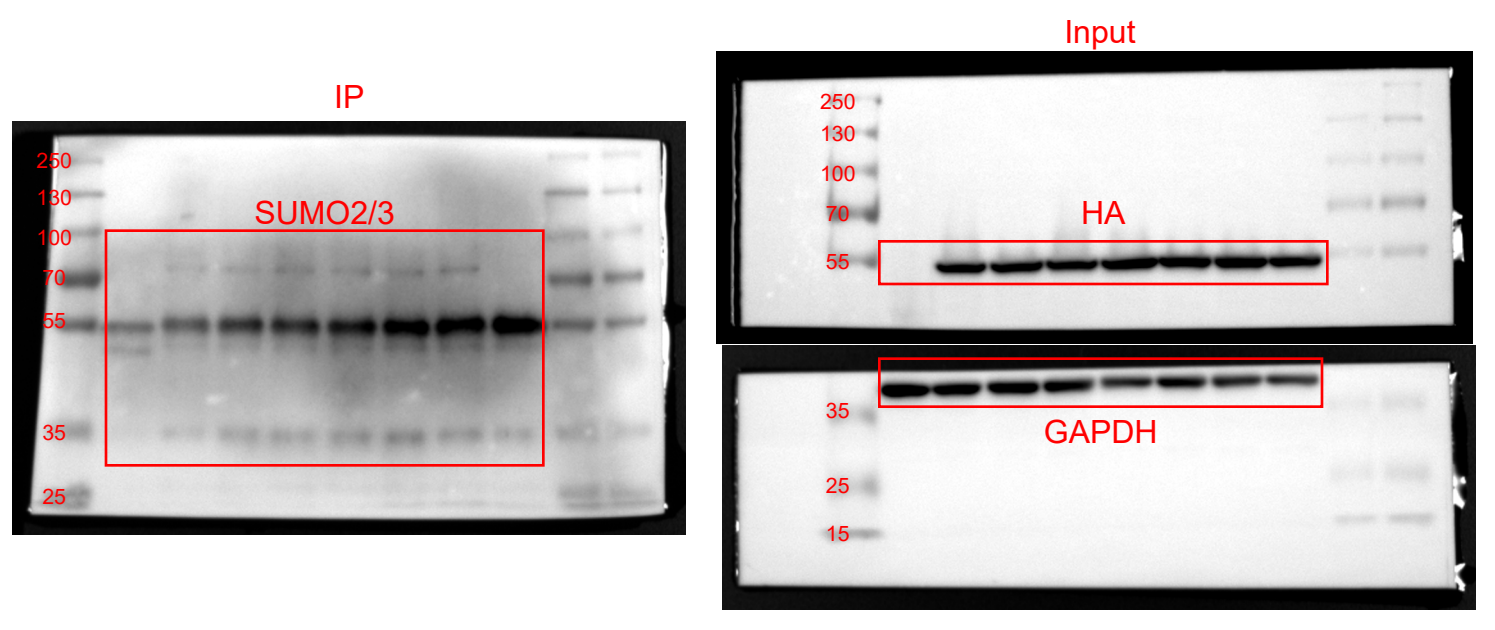

Fig. 4H
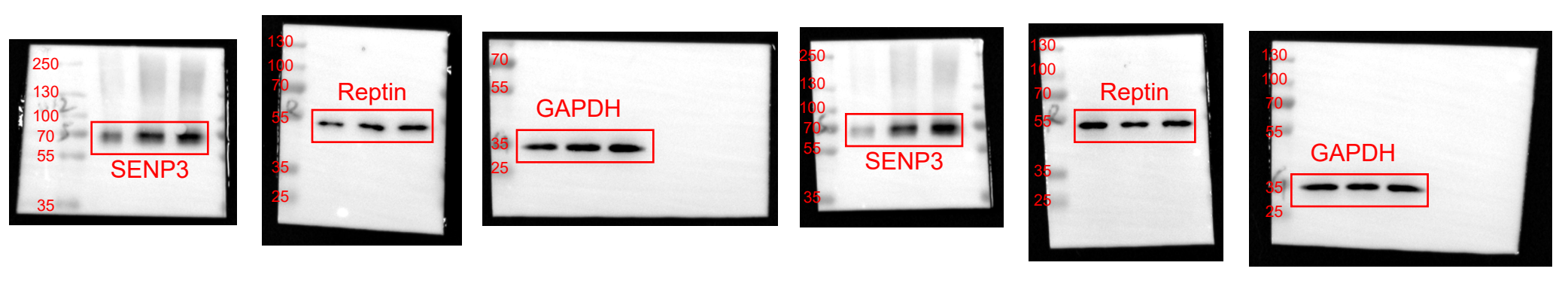

Fig. 4I
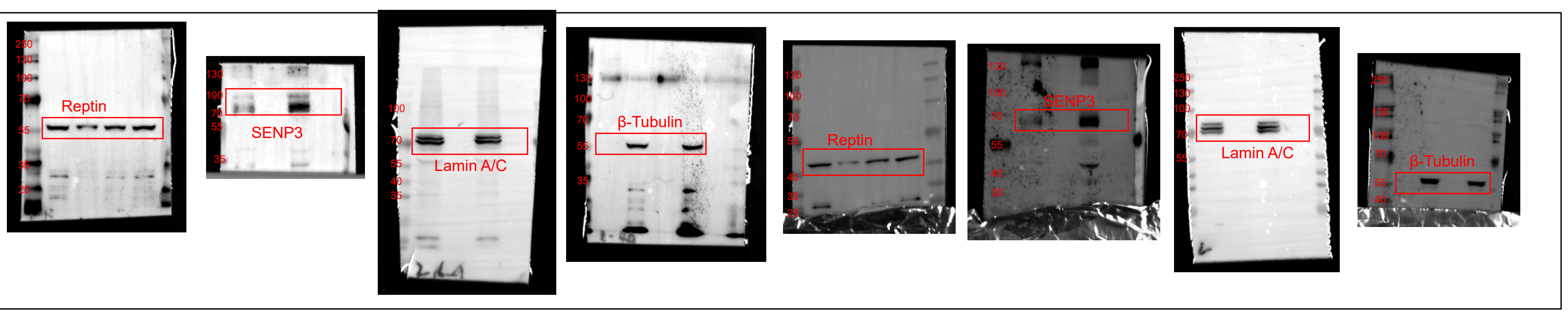

Fig. 4K
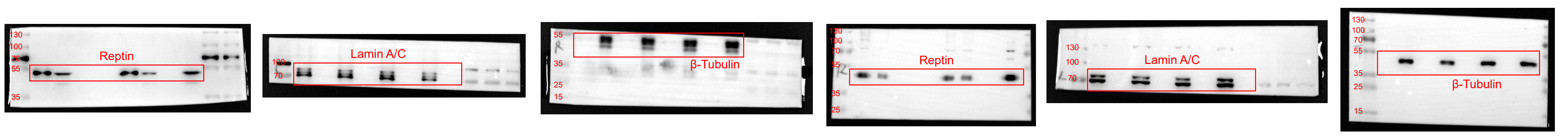

Fig. 5G
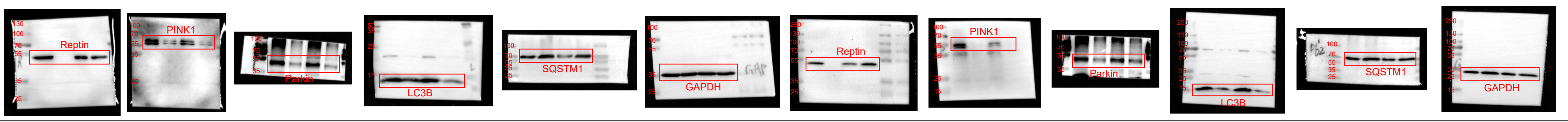

Fig. 6A
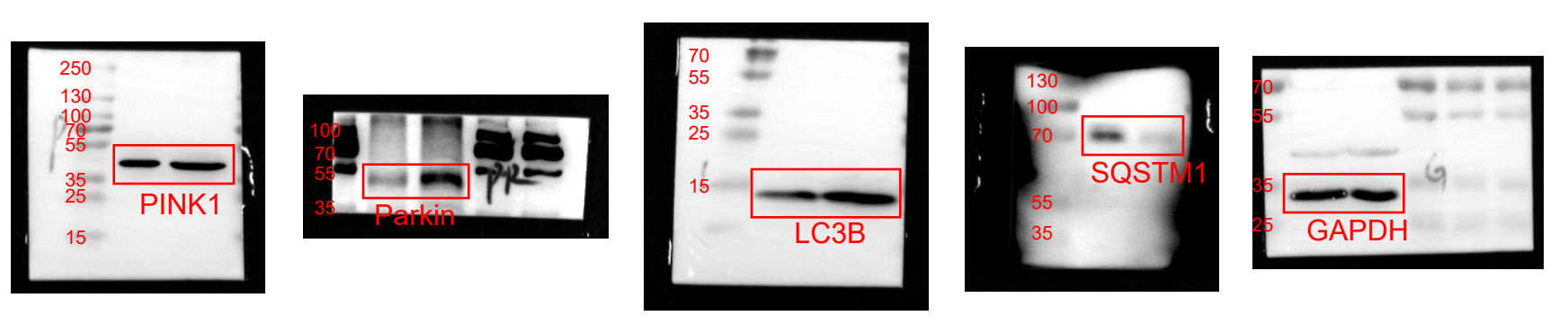

Fig. 6G
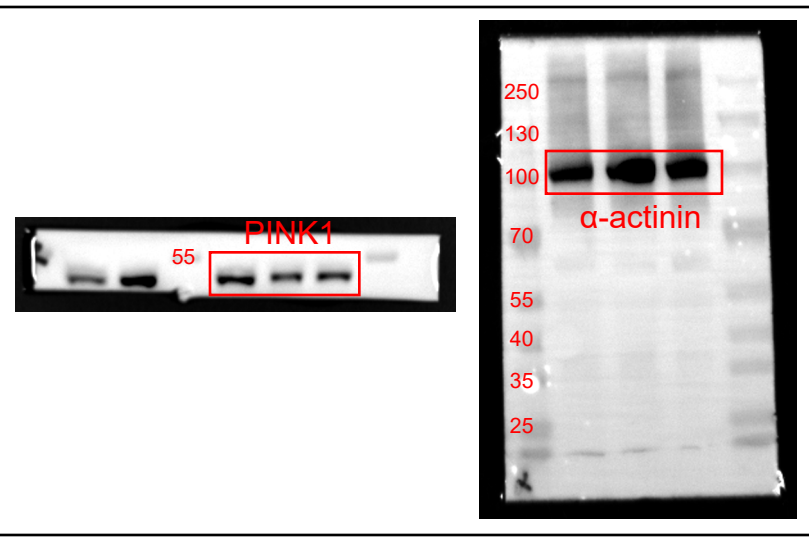

Fig. 7F
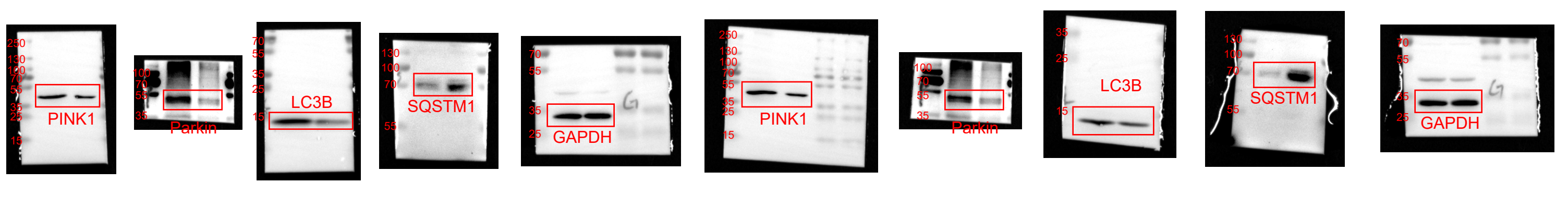

Fig. S1A
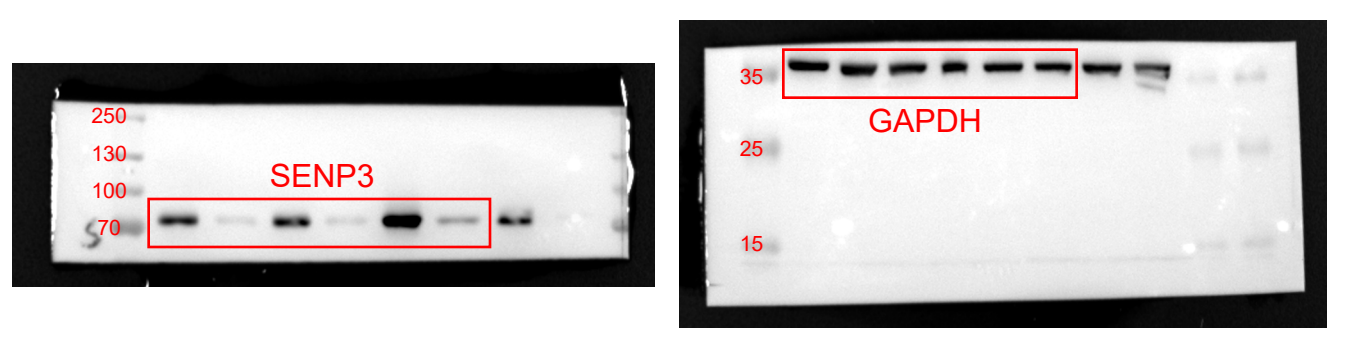

Fig. S1B
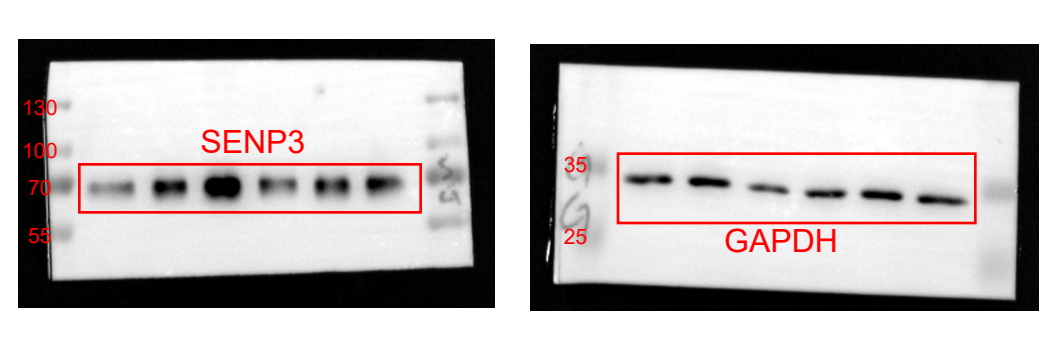

Fig. S1C
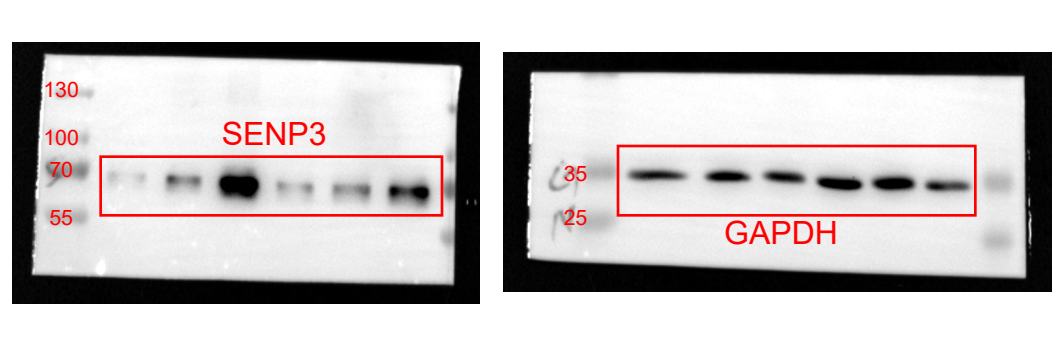

Fig. S1E
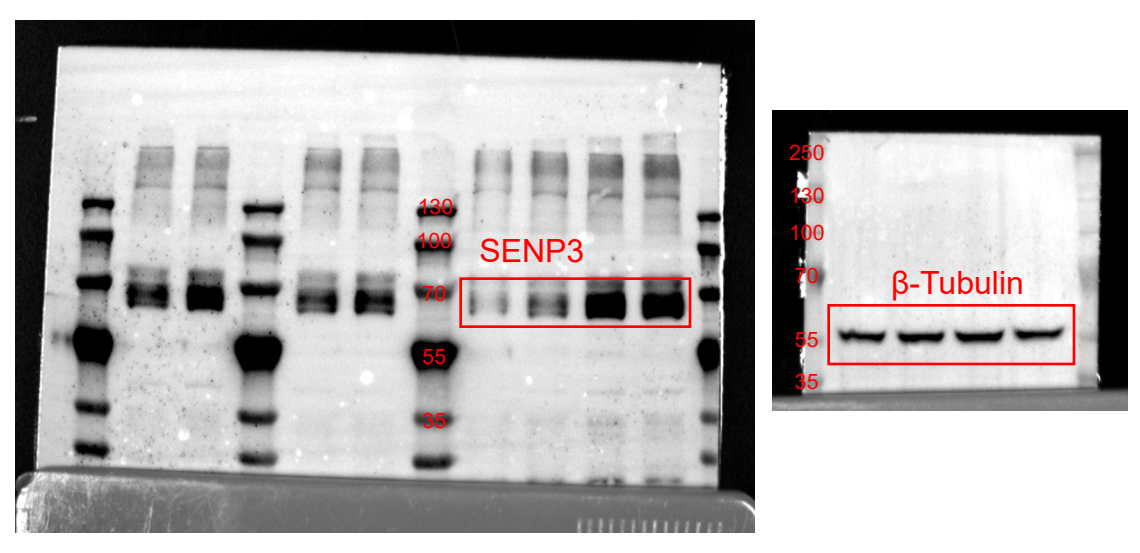

Fig. S2C
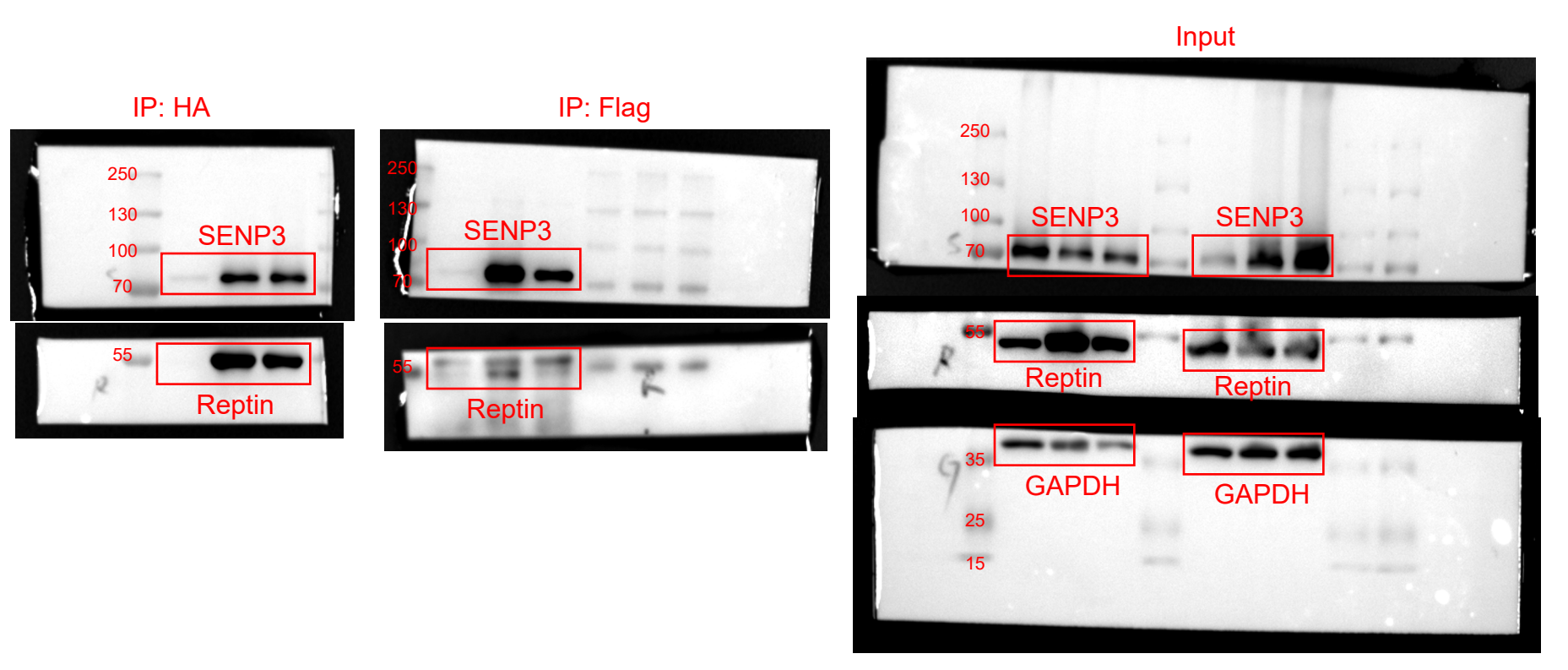

Fig. S2D
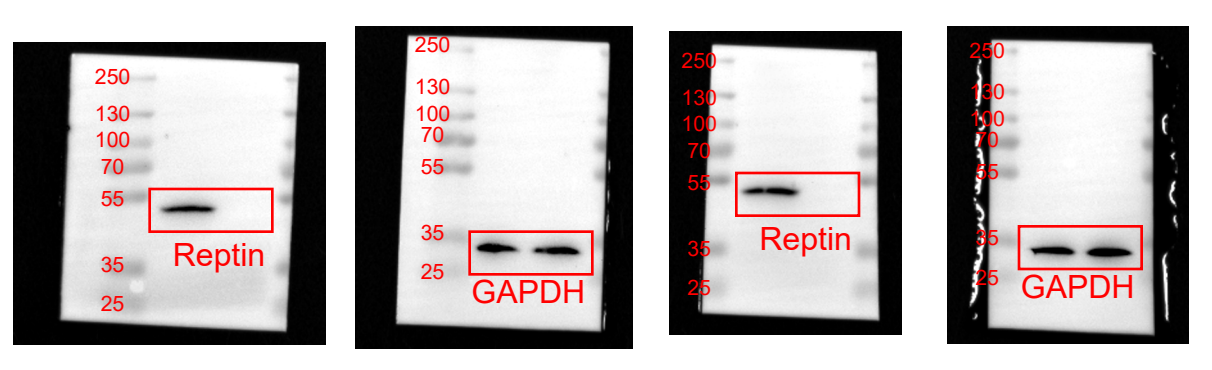

Fig. S3G
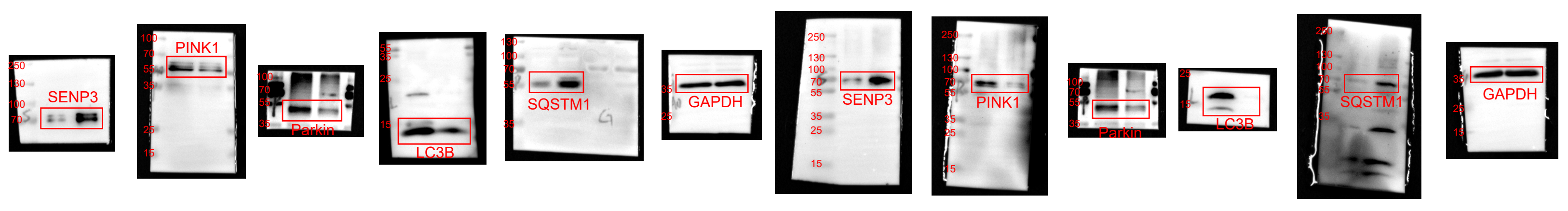

Supplement: Supplementary file 1 [file 41419_2026_8598_MOESM7_ESM.pdf]
