# Supplementary material for: SUMOylated Reptin maintains low intracellular ROS levels by activating mitophagy to drive gemcitabine resistance in gallbladder cancer
Source: Cell Death Dis. 2026 Sep 10;17(1):394. doi: 10.1038/s41419-026-08598-0 (PMC13558568; doi:10.1038/s41419-026-08598-0)
Supplement: Supplementary file 3 [file 41419_2026_8598_MOESM1_ESM.docx]

**Supplementary Table 1. Clinical information on GBC patients received gemcitabine-based therapy after surgery (Cohort 1).**

| No. | Age | Gender | Tumor grade | Tumor size | AJCC stage^a^ | SENP3 | Reptin | OS (months) | Status |
| --- | --- | --- | --- | --- | --- | --- | --- | --- | --- |
| 1 | 63 | Female | III-IV | <4 cm | III-IV | high | high | 12.1 | dead |
| 2 | 61 | Female | I-II | <4 cm | I-II | high | high | 131.8 | live |
| 3 | 42 | Female | III-IV | <4 cm | III-IV | low | low | 8.4 | dead |
| 4 | 58 | Female | I-II | <4 cm | III-IV | low | low | 111.8 | dead |
| 5 | 73 | Female | I-II | <4 cm | I-II | low | low | 14.5 | dead |
| 6 | 69 | Male | I-II | <4 cm | I-II | low | low | 124.3 | live |
| 7 | 73 | Female | I-II | ≥4 cm | III-IV | low | high | 3.2 | dead |
| 8 | 51 | Male | III-IV | <4 cm | III-IV | low | high | 10.5 | dead |
| 9 | 75 | Male | I-II | <4 cm | I-II | high | low | 108.4 | live |
| 10 | 68 | Female | I-II | <4 cm | I-II | low | low | 64.9 | dead |
| 11 | 62 | Female | I-II | <4 cm | I-II | high | high | 28.3 | live |
| 12 | 52 | Male | I-II | ≥4 cm | I-II | low | high | 7.6 | dead |
| 13 | 75 | Female | III-IV | ≥4 cm | III-IV | low | low | 12.7 | dead |
| 14 | 83 | Female | I-II | ≥4 cm | III-IV | high | high | 18.5 | dead |
| 15 | 60 | Male | I-II | ≥4 cm | III-IV | high | high | 3.1 | dead |
| 16 | 75 | Male | I-II | ≥4 cm | I-II | low | low | 26.3 | dead |
| 17 | 70 | Female | III-IV | ≥4 cm | III-IV | low | low | 12.0 | dead |
| 18 | 67 | Male | I-II | ≥4 cm | III-IV | low | high | 8.3 | dead |
| 19 | 50 | Male | III-IV | <4 cm | III-IV | low | high | 3.0 | dead |
| 20 | 48 | Female | I-II | ≥4 cm | III-IV | high | high | 7.9 | dead |
| 21 | 75 | Female | I-II | <4 cm | III-IV | low | high | 5.1 | dead |
| 22 | 75 | Female | III-IV | <4 cm | III-IV | low | low | 20.8 | dead |
| 23 | 50 | Male | I-II | <4 cm | III-IV | low | low | 9.1 | dead |
| 24 | 75 | Male | III-IV | ≥4 cm | III-IV | high | high | 13.2 | dead |
| 25 | 65 | Female | I-II | <4 cm | III-IV | low | high | 10.2 | dead |
| 26 | 57 | Female | I-II | ≥4 cm | III-IV | high | high | 17.9 | dead |
| 27 | 71 | Female | III-IV | ≥4 cm | III-IV | low | low | 6.7 | dead |
| 28 | 65 | Male | III-IV | ≥4 cm | III-IV | high | high | 4.5 | dead |
| 29 | 59 | Male | I-II | <4 cm | III-IV | high | low | 26.5 | dead |
| 30 | 79 | Female | III-IV | <4 cm | III-IV | low | low | 10.2 | dead |
| 31 | 69 | Female | III-IV | ≥4 cm | III-IV | low | high | 16.3 | dead |
| 32 | 72 | Female | III-IV | ≥4 cm | III-IV | low | high | 8.0 | dead |
| 33 | 62 | Male | I-II | <4 cm | III-IV | high | low | 31.1 | dead |
| 34 | 72 | Female | I-II | ≥4 cm | III-IV | high | high | 2.0 | dead |
| 35 | 63 | Female | III-IV | ≥4 cm | III-IV | high | high | 26.2 | dead |
| 36 | 54 | Female | I-II | ≥4 cm | III-IV | low | low | 14.5 | dead |
| 37 | 62 | Female | I-II | ≥4 cm | III-IV | high | high | 8.5 | dead |
| 38 | 48 | Male | I-II | ≥4 cm | III-IV | high | low | 64.3 | live |
| 39 | 80 | Female | III-IV | <4 cm | III-IV | high | high | 35.9 | dead |
| 40 | 58 | Male | III-IV | ≥4 cm | III-IV | low | high | 3.5 | dead |
| 41 | 83 | Female | I-II | ≥4 cm | III-IV | high | high | 11.2 | dead |
| 42 | 59 | Female | I-II | <4 cm | III-IV | low | low | 24.1 | dead |
| 43 | 63 | Female | I-II | <4 cm | III-IV | low | low | 10.8 | dead |
| 44 | 56 | Female | I-II | <4 cm | III-IV | high | low | 24.0 | dead |
| 45 | 57 | Female | I-II | ≥4 cm | III-IV | high | high | 50.8 | live |
| 46 | 79 | Female | III-IV | ≥4 cm | III-IV | low | high | 8.7 | dead |
| 47 | 69 | Female | III-IV | ≥4 cm | III-IV | low | low | 11.1 | dead |
| 48 | 65 | Female | I-II | <4 cm | III-IV | low | high | 6.8 | dead |
| 49 | 80 | Male | I-II | <4 cm | I-II | low | low | 16.7 | dead |
| 50 | 63 | Male | I-II | ≥4 cm | III-IV | high | high | 12.6 | dead |
| 51 | 56 | Female | I-II | <4 cm | I-II | low | low | 29.9 | dead |
| 52 | 72 | Female | III-IV | <4 cm | III-IV | high | high | 18.3 | dead |
| 53 | 70 | Male | I-II | ≥4 cm | I-II | low | high | 10.3 | dead |
| 54 | 60 | Female | III-IV | ≥4 cm | III-IV | low | low | 6.9 | dead |
| 55 | 83 | Male | I-II | ≥4 cm | III-IV | low | high | 3.8 | dead |
| 56 | 79 | Male | I-II | <4 cm | III-IV | low | high | 3.2 | dead |
| 57 | 76 | Female | I-II | ≥4 cm | III-IV | low | low | 14.1 | dead |
| 58 | 71 | Male | III-IV | ≥4 cm | III-IV | low | high | 8.8 | dead |
| 59 | 60 | Female | I-II | <4 cm | I-II | high | high | 26.4 | dead |
| 60 | 59 | Female | III-IV | ≥4 cm | III-IV | high | high | 12.5 | dead |
| 61 | 75 | Female | III-IV | ≥4 cm | III-IV | low | high | 3.0 | dead |
| 62 | 59 | Female | I-II | ≥4 cm | III-IV | low | high | 11.1 | dead |

^a^AJCC stage, based on the American Joint Committee in Cancer (AJCC)/International Union Against Cancer staging manual (8th edition). OS, overall survival.

**Supplementary Table 2. Clinical information on GBC patients received gemcitabine-based therapy after surgery (Cohort 2).**

| No. | Age | Gender | SENP3 | Reptin | OS (months) | Status |
| --- | --- | --- | --- | --- | --- | --- |
| 1 | 62 | Female | high | low | 14.4 | dead |
| 2 | 72 | Female | high | high | 7.2 | dead |
| 3 | 59 | Female | high | low | 40.3 | dead |
| 4 | 57 | Male | high | low | 25.2 | dead |
| 5 | 58 | Male | high | low | 15.5 | dead |
| 6 | 74 | Female | low | low | 21.2 | dead |
| 7 | 51 | Female | high | low | 50.4 | live |
| 8 | 69 | Male | high | low | 34.8 | dead |
| 9 | 76 | Female | low | high | 24 | dead |
| 10 | 61 | Male | low | low | 19.1 | dead |
| 11 | 52 | Female | low | high | 10.8 | dead |
| 12 | 66 | Male | high | low | 25.2 | dead |
| 13 | 87 | Female | low | high | 14.7 | dead |
| 14 | 72 | Female | low | high | 4.8 | dead |
| 15 | 47 | Female | low | high | 2.4 | dead |
| 16 | 61 | Male | high | high | 6 | dead |
| 17 | 55 | Male | low | low | 12.1 | dead |
| 18 | 79 | Female | low | high | 4.8 | dead |
| 19 | 79 | Male | low | high | 3.5 | dead |
| 20 | 83 | Female | high | low | 45.2 | dead |
| 21 | 77 | Female | low | high | 8.6 | dead |
| 22 | 60 | Female | high | low | 9.7 | dead |
| 23 | 61 | Female | low | high | 18 | dead |
| 24 | 67 | Male | high | low | 39.1 | dead |
| 25 | 77 | Female | high | low | 21.5 | dead |
| 26 | 55 | Female | low | low | 18.6 | dead |
| 27 | 65 | Female | low | low | 8.4 | dead |
| 28 | 61 | Male | low | high | 6 | dead |
| 29 | 57 | Female | low | low | 9.6 | dead |
| 30 | 79 | Female | low | high | 12 | dead |
| 31 | 68 | Female | high | high | 14.4 | dead |
| 32 | 65 | Female | low | high | 14.1 | dead |
| 33 | 45 | Male | high | low | 43.2 | live |
| 34 | 78 | Female | low | high | 8.4 | dead |
| 35 | 77 | Female | low | high | 1.2 | dead |

OS, overall survival.

**Supplementary Table 3. Clinical information on GBC patients received mini-PDX model test (Cohort 3).**

| No. | AJCC stage^a^ | Gemcitabine viability (%) | SENP3 | Reptin |
| --- | --- | --- | --- | --- |
| 1 | IIIb | 134 | low | high |
| 2 | IVa | 112 | low | high |
| 3 | IVb | 103 | low | low |
| 4 | IIIb | 103 | low | high |
| 5 | IVb | 94 | low | low |
| 6 | IVa | 86 | low | high |
| 7 | IVb | 82 | high | high |
| 8 | IIIb | 77 | low | high |
| 9 | IIIb | 70 | low | high |
| 10 | IIIa | 68 | high | low |
| 11 | IVb | 67 | high | low |
| 12 | IVa | 64 | high | low |
| 13 | IVb | 58 | high | low |
| 14 | IIIa | 55 | high | low |
| 15 | IIIb | 47 | high | low |
| 16 | IVb | 46 | high | low |
| 17 | IVa | 42 | high | low |
| 18 | IVb | 40 | high | low |

^a^AJCC stage, based on the American Joint Committee in Cancer (AJCC)/International Union Against Cancer staging manual (8th edition).

**Supplementary Table 4. List primers used in this study.**

| Real time PCR primer | Forward (5'-3') | Reverse (5'-3') |
| --- | --- | --- |
| 18S  SENP3  PINK1  BNIP3  BNIP3L  FUNDC1  PINK1 promoter | TTCGAACGTCTGCCCTATCAA  GCACTGATGAGGTAGTAGAGAAGC  GCCTCATCGAGGAAAAACAGG  AGCGTTCCAGCCTCGGTTTC  AACAACAACAACAACTGCGAGGAA  TGGCGTTACTGGCTGGTGTG  GCCGCTGAAGCCAGAGACTAT | ATGGTAGGCACGGCGACTA  TGGCATCCGCTGGTAAGACT  GTCTCGTGTCCAACGGGTC  GCTCTGTTGGTATCTTGTGGTGTC  TGTGGATGGAGGATGAGGATGGTA  TGCTGCTTTGTTCGCTCGTTTC  CACGCACGACACGCACATT |
| sgRNA sequence | Sense (5'-3') | Antisense (5'-3') |
| sgReptin | CACCAGATTGATCGACCAGCAACA | AAACTGTTGCTGGTCGATCAATCT |
| siRNA sequence | Sense (5'-3') | Antisense (5'-3') |
| siPINK1-1  siPINK1-2 | CCGCAAAUGUGCUUCAUCUAATT  AUGGGUCAGCACGUUCAGUUATT | TTGGCGUUUACACGAAGUAGAUU  TTUACCCAGUCGUGCAAGUCAAU |
| shRNA sequence | | |
| shSENP3-1-F  shSENP3-1-R  shSENP3-2-F  shSENP3-2-R | 5'-CCGGCCGGATGCTGCTCTACTCAAACTCGAGTTTGAGTAGAGCAGCATCCGGTTTTTG-3'  5'-AATTCAAAAACCGGATGCTGCTCTACTCAAACTCGAGTTTGAGTAGAGCAGCATCCGG-3'  5'-CCGGCCTCGCTGACATTCCACTGGACTCGAGTCCAGTGGAATGTCAGCGAGGTTTTTG-3'  5'-AATTCAAAAACCTCGCTGACATTCCACTGGACTCGAGTCCAGTGGAATGTCAGCGAGG-3' | |
