# Supplementary material for: SUMOylated Reptin maintains low intracellular ROS levels by activating mitophagy to drive gemcitabine resistance in gallbladder cancer
Source: Cell Death Dis. 2026 Sep 10;17(1):394. doi: 10.1038/s41419-026-08598-0 (PMC13558568; doi:10.1038/s41419-026-08598-0)
Supplement: Supplementary file 4 [file 41419_2026_8598_MOESM6_ESM.docx]

**Supplementary Figure 1. SENP3 responds to changes in ROS levels in GBC cells but does not affect their basal proliferation or migration.** (A) Western blot analysis after 72 h of treatment with 4 μM gemcitabine. SENP3 protein levels in gemcitabine-resistant GBC cell line (GEM-R) are significantly lower than in wild-type (WT) NOZ cells. (B-C) Western blot analysis of the effect of H_2_O_2_ and NAC on SENP3 protein levels. (B) GEM-R cells; (C) WT NOZ cells. Treatment with different concentrations of H_2_O_2_ for 1 h increases intracellular SENP3 protein levels in a concentration-dependent manner. This effect is antagonized by pre-treatment with 5 mM NAC for 4 h. (D) RT-qPCR analysis. Elevation of ROS levels does not affect SENP3 mRNA levels. (E) Western blot analysis. SENP3 protein levels were evaluated after NOZ cells were treated with H_2_O_2_ as indicated for 1 h with or without 30 mM MG132. (F) Quantitative analysis of Western blots in Figure 2C. (G) Cell viability assays (0-96 h). SENP3-overexpression has no significant effect on the proliferative capacity of NOZ and GBC-SD cells. (H) Cell migration assays. SENP3-overexpression has no significant effect on the migratory capacity of NOZ and GBC-SD cells. Scale bar: 50 μm. Data represent the mean ± SEM (n = 3 independent experiments). Student’s t-tests (A), one-way ANOVA (B, C, D, E, F), two-way ANOVA (G), and Mann-Whitney U test (H) were used.

**Supplementary Figure 2. SENP3 deSUMOylates Reptin at the K456 site.** (A) List of the top 8 most abundant interacting proteins identified by mass spectrometry analysis of SENP3 co-immunoprecipitation (co-IP) products. (B) Prediction of potential SUMOylation sites in Reptin protein (SUMOplot/GPS-SUMO). Six candidate lysine sites are labeled in red (K115, K132, K269, K288, K368, K456). (C) Exogenous co-IP to verify the effect of Reptin K456R mutation on binding. Western blot of 293T cells transfected with indicated plasmids for 72 h confirms that, compared with wild-type Reptin, the K456R mutant shows unaltered binding affinity to SENP3. (D) Western blot validation of efficient Reptin knockout (RKO) by CRISPR-Cas9 in NOZ and GBC-SD cells.

**Supplementary Figure 3. SUMOylated Reptin promotes mitophagy through transcriptional activation of PINK1 (supplementary data).** (A) Cell viability assays: in Reptin-knockout (RKO) NOZ/GBC-SD cells, rescue with WT Reptin significantly increases the gemcitabine IC50, while the K456R mutant has no effect. (B) Schematic of ChIP-seq peak distribution in the transcription start site (TSS) region of the PINK1 gene (6 significant peaks detected). (C) RT-qPCR analysis after 48 h of gemcitabine treatment: (C) SENP3-overexpression or (D) Reptin-knockout does not alter mRNA levels of BNIP3, BNIP3L, or FUNDC1. (E) In the presence of the antioxidant NAC to maintain stable ROS, overexpression of SENP3 still reduces PINK1 mRNA levels. (F) ChIP-qPCR analysis in GBC-SD cells. Knockdown of Reptin reduces its enrichment at the PINK1 promoter. This enrichment is restored by reconstitution with WT Reptin, but not with the K456R SUMOylation-deficient mutant. (G) Western blot after 72 h of gemcitabine treatment shows that SENP3-overexpression reduces levels of mitophagy-related proteins in NOZ and GBC-SD cells. (H) Quantitative analysis of Western blots in Sup Figure 5G. (I) Quantitative analysis of Western blots in Figure 5G. Data represent the mean ± SEM (n = 3 independent experiments). Two-way ANOVA (A), Student’s t-tests (C, D, E, H), and one-way ANOVA (F, I) were used.

**Supplementary Figure 4. CB-6644 specifically targets SUMOylated Reptin and exhibits favorable pharmacokinetics and safety profiles *in vivo*.** (A) Quantitative analysis of Western blots in Figure 7F. (B) Quantitative analysis of Figure 7G. (C) Quantitative analysis of Figure 7H. (D) Quantitative analysis of Figure 7I. (E) Cell viability assays in Reptin-knockout (RKO) cells demonstrate that CB-6644 resensitizes cells reconstituted with wild-type (WT) Reptin to gemcitabine, but has no effect on cells reconstituted with the SUMOylation-deficient K456R mutant. This confirms the specificity of CB-6644 for the SUMOylated form of Reptin. (F) Schematic of the xenograft tumor experiment in NOD-Scid mice. 2×10⁶ NOZ or GBC-SD cells were injected subcutaneously into the right axilla of NOD-Scid mice. Data represent the mean ± SEM (n = 3 independent experiments). (G) Plasma concentration-time profile of CB-6644 after a single intraperitoneal injection in mice, showing rapid clearance within 24 h. (H) Tissue distribution analysis reveals higher concentrations of CB-6644 in healthy organs (particularly liver and spleen) compared to tumor tissue post-injection. (I) Representative H&E staining of liver and kidney sections from treated mice shows no apparent histopathological damage. Scale bar: 100 μm (J) Serum levels of alanine aminotransferase (ALT) and aspartate aminotransferase (AST) show no significant elevation in CB-6644-treated groups compared to controls, indicating no acute liver toxicity. Data represent the mean ± SEM. Student’s t-tests (A, B, C, D), two-way ANOVA (E), and one-way ANOVA (J) were used.
